# Diagnostic and prognostic values of differentially expressed genes in canine mammary carcinoma: An integrated bioinformatics analysis

**DOI:** 10.1371/journal.pone.0354033

**Published:** 2026-07-20

**Authors:** Oscar Hernán Rodríguez-Bejarano, David Santiago Padilla, Daniel Alzate, Lucía Botero, Giovanni Vargas Hernández, Liliana López-Kleine, Manuel Alfonso Patarroyo, Carlos A. Parra-Lopez

**Affiliations:** 1 Health Sciences Faculty, Universidad de Ciencias Aplicadas y Ambientales (U.D.C.A), Bogotá, Colombia; 2 Molecular Biology and Immunology Department, Fundación Instituto de Inmunología de Colombia (FIDIC), Bogotá, Colombia; 3 PhD Programme in Biotechnology, Faculty of Sciences, Universidad Nacional de Colombia, Bogotá, Colombia; 4 Immunology and Translational Medicine Group, Faculty of Medicine, Universidad Nacional de Colombia, Bogotá, Colombia; 5 Faculty of Veterinary Medicine and Zootechnics, Universidad Nacional de Colombia, Carrera, Bogotá, Colombia; 6 Clínica de Pequeños Animales Universidad Nacional de Colombia (CPA-UN), Bogotá, Colombia; 7 Statistics Department, Faculty of Sciences, Universidad Nacional de Colombia, Bogotá, Colombia; 8 Microbiology Department, Faculty of Medicine, Universidad Nacional de Colombia, Bogotá, Colombia; OMICS, PERU

## Abstract

**Background:**

Canine mammary carcinoma (CMC) is a common tumor in unspayed dogs and poses a significant health concern for animals. This study aimed to identify differentially expressed genes (DEGs) between CMC and adjacent healthy mammary tissue through an integrated RNASeq bioinformatics analysis that combined an independently generated dataset from the present study, CPA-UN (CPA-UN), composed of RNA-seq data obtained from CMC and matched normal mammary tissue samples, with publicly available GEO datasets generated using next-generation sequencing (NGS). Candidate genes associated with diagnostic and prognostic potential were subsequently explored through integrative downstream analyses.

**Methods and findings:**

DEGs were identified using DESeq2 and further analyzed for functional enrichment (ClusterProfiler, Pathview, and GSEA), co-expression gene networks, and tumor immune infiltrate deconvolution (CIBERSORTx). The GSE119810 dataset was used for exploratory overall survival (OS) analysis. Transcriptomic data from 88 CMC cases and adjacent healthy mammary tissue samples identified eight DEGs common across all four datasets. Among these, *ACAN*, *COL11A1*, *EDIL3*, *NDUFA4L2*, and *IGFBP5* showed higher transcript levels in CMC tissues than in healthy mammary tissue, whereas *TNNC1*, *PCK1*, and *METTL24* showed lower transcript levels in CMC tissues than in healthy mammary tissue. Functional enrichment analyses indicated that DEGs with higher transcript levels in CMC were predominantly associated with extracellular matrix remodeling, cell adhesion, tumor microenvironment interactions, immune and inflammatory responses, and signaling pathways involved in tumor progression and metastasis. In contrast, DEGs with lower transcript levels were mainly enriched for cytoskeletal organization and tissue structural integrity, suggesting a loss of normal mammary gland architecture and myoepithelial-associated functions during tumor progression. GSEA further demonstrated coordinated enrichment of hallmark gene sets related to cell-cycle dysregulation, proliferation, epithelial–mesenchymal transition, metabolic adaptation, inflammatory signaling, and stromal remodeling, supporting the presence of integrated transcriptional programs that drive tumor progression and TME remodeling in CMC. Co-expression gene network analysis revealed a highly modular organization, with densely interconnected clusters of co-expressed genes that may reflect coordinated biological processes and regulatory programs. Exploratory CIBERSORTx analysis found no significant differences in the relative proportions of the 22 infiltrating immune cell types between CMCs and paired healthy controls after multiple-comparison corrections. Nevertheless, dataset-specific trends were observed in regulatory T cells, M1 macrophages, activated CD4 memory T cells, plasma cells, dendritic cells, and mast cells, though these findings should be interpreted cautiously. An exploratory survival analysis of 1,759 DEGs from the GSE119810 dataset identified 53 genes nominally associated with overall survival in CMC. However, none remained statistically significant after multiple-testing correction, underscoring the exploratory nature of these findings and the need for validation in larger cohorts.

**Conclusion:**

This study provides a preliminary transcriptomic framework for CMC, identifying candidate genes and pathways associated with tumor-related processes and supporting future functional validation to clarify their roles in tumorigenesis, progression, and tumor aggressiveness. However, these findings remain exploratory and require validation in larger cohorts to confirm their diagnostic and prognostic relevance, given the limitations of secondary data analyses and potential variability in tissue collection and processing across studies.

## Introduction

Mammary tumors are a common type of neoplasia in unspayed adult dogs and pose a major health concern. About 50% of all tumors in dogs are mammary tumors, and their prevalence is nearly three times higher in females. Approximately 45% of mammary tumors are canine mammary carcinomas (CMC) [1]. Recent studies have shown an increase in CMC cases relative to benign tumors, following a trend similar to that observed in human medicine [2–4]. The prognosis for CMC mainly depends on the histological grade (grade I, II, or III) and the clinical stage (T (tumor size), N (nodal involvement), M (distant metastasis); TNM system approved by the WHO), with higher grades of malignancy and more advanced stages associated with poorer outcomes [5–8]. CMC-related mortality remains relatively high, with more than 40% of dogs dying within one year of diagnosis [9]. Domestic dogs and humans are exposed to similar environmental conditions and may share lifestyle-related risk factors, underscoring the relevance of comparative oncology approaches within the One Health framework [9,10].

CMC has been proposed as a comparative model for human breast cancer (HBC) because both diseases share several epidemiological, clinical, histopathological, hormonal, and molecular characteristics, including spontaneous tumor development, age-associated incidence, hormone-related influences, and similar gene expression alterations and biological behavior [11,12]. In addition, domestic dogs and humans are exposed to comparable environmental conditions and may share lifestyle-associated risk factors, supporting the relevance of comparative oncology approaches within the One Health framework [9,10]. Using the canine biomodel as a translational animal model for HBC research could improve understanding of tumor biology and aid in the discovery of biomarkers useful for both species [13].

Although some transcriptomic studies on CMC have been reported, many remain limited by relatively small cohorts, single-dataset designs, and analyses focused primarily on differential gene expression [14–17]. Consequently, consistent transcriptional alterations associated with CMC across independent studies remain poorly characterized, and integrative transcriptomic analyses in this tumor type are still limited. To address these gaps, the present study integrated multiple independent RNASeq datasets and analyzed them using a comprehensive bioinformatic approach.

Accordingly, this study aimed to identify differentially expressed genes (DEGs) between CMC and adjacent healthy mammary tissue through an integrated RNASeq analysis that combined an independently generated dataset from the present study (CPA-UN), comprising RNASeq data from CMC and matched normal mammary tissue samples, with publicly available Gene Expression Omnibus (GEO) datasets generated using next-generation sequencing (NGS). Candidate genes with potential diagnostic and prognostic relevance were subsequently explored through integrative downstream analyses. Nevertheless, given the exploratory nature of this study, further validation in larger and independent cohorts, as well as functional and mechanistic studies, will be necessary to confirm the biological and clinical relevance of the identified candidates.

## Materials and methods

### Ethical approval and consent to participate

The Institutional Committee for the Care and Use of Animals in Research and Teaching (CICUA) at the Faculty of Animal Sciences, Universidad de Ciencias Aplicadas y Ambientales (U.D.C.A), Bogotá, Colombia, requested approval of the study design and protocol, in accordance with the policy and guidelines for ethical conduct in animal care and use (Minutes 29-01-2024). Informed consent was obtained from the owners of the dogs involved in this study, and strict confidentiality of both the owners and the animals was maintained.

### Cases and specimens

Twelve canine specimens with suspected CMC were included in this study. They underwent radical mastectomy as part of the standard treatment protocol at the “Clínica de Pequeños Animales Universidad Nacional (CPA-UN)”. During surgery, the veterinary surgeon collected an incisional sample exclusively from the tumor region careful dissection to avoid including surrounding skin or muscle tissue. In addition, a sample of the adjacent normal mammary gland was obtained from the mastectomy specimen for comparative analyses. Tissue fragments were trimmed to approximately 0.5 cm in each dimension and immediately immersed in RNAlater solution (Invitrogen, Waltham, MA, USA) at a ratio of about five volumes of solution per unit of tissue mass, following the manufacturer’s recommendations. Samples were kept at 4°C overnight and then stored at −70°C until processing. The remaining tissue was fixed in 10% buffered formalin and processed for routine histopathological diagnosis (H&E staining) at the CPA-UN Pathology Laboratory. Histopathological classification and tumor grade were determined by two independent veterinary pathologists using the classification of Goldschmidt et al. and the Nottingham histological grading parameters for CMC (ca-NHG) [7]. A histological examination of the regional lymph node was also conducted to assess for metastatic involvement. Medical history was reviewed to collect clinical data including breed, age, tumor size, and imaging assessments for distant metastases (chest X-ray and abdominal ultrasound). Clinical staging was performed according to the TNM system [8]. Two of the recruited cases were excluded because the histopathological diagnosis was mammary adenoma. The clinical characteristics of the 10 canine specimens analyzed are listed in Table 1.

**Table 1:** Clinical data of canines with mammary carcinoma used for analysis.

Case	Breed	Age (years)	Diagnosis	Grade	Lymph Node Metastasis	Distant Metastasis	Clinical Stage (TNM)
DMV	French Poodle	10	Complex carcinoma	I	No	No	I (T1N0M0)
RON	Labrador Retriever	11	Complex carcinoma	I	No	No	I (T1N0M0)
EGD	Labrador Retriever	11	Complex carcinoma	II	No	No	I (T1N0M0)
PAR	Beagle	12	Simple carcinoma	III	Yes	No	IV (T3N1M0)
NDD	Mixed	9	Complex carcinoma	I	No	No	III (T3N0M0)
MAR	Mixed	11	Complex carcinoma	I	No	No	II (T2N0M0)
EPG	Mixed	10	Simple carcinoma	I	No	No	II (T2N0M0)
SMA	Mixed	9	Simple carcinoma	I	No	No	II (T2N0M0)
OAM	Dalmatian	12	Simple carcinoma	I	No	No	II (T2N0M0)
ACC	Boston Terrier	8	Simple carcinoma	I	No	No	II (T2N0M0)

### RNA isolation and sequencing

Total RNA was extracted from CMCs and adjacent healthy mammary tissue using the RNeasy Mini Plus kit (Qiagen, Valencia, CA, USA). Samples were homogenized by liquid nitrogen pulverization before RNA isolation, following the manufacturer’s instructions. RNA quality was assessed by analyzing the integrity of 18S and 28S ribosomal RNA (rRNA) bands with the Agilent RNA 6000 Nano kit (part # 5067-1511) on an Agilent 2100 Bioanalyzer (Agilent Technologies, Santa Clara, CA, USA). Messenger RNA was purified from total RNA using oligonucleotide-linked poly-T magnetic beads. After fragmentation, first-strand cDNA was synthesized using random hexamer primers. Then, the second strand of cDNA was synthesized using dUTP instead of dTTP with the TruSeq Stranded Total RNA Sample Preparation Kit (RS-122-9007) (Illumina, San Diego, CA, USA). The directional library was prepared after end repair, A-tailing, adapter ligation, size selection, USER digestion, amplification, and purification. The library was analyzed for size distribution using an Agilent DNA 1000 Kit (part # 5067-1504) and an Agilent 2100 Bioanalyzer (Agilent Technologies, Santa Clara, CA, USA), and quantified using a Qubit 2100 fluorometer (Thermo Scientific, Waltham, MA, USA) and real-time PCR. Libraries were pooled based on effective library concentration and data volume and sequenced on Illumina platforms, including the Illumina NovaSeq X Plus (PE 150). Raw data quality was assessed using FastQC (version 0.74) [18]. A summary of the RNA sequencing statistics is included in S1 Table. Our dataset was named “CPA-UN” and the corresponding raw RNASeq data are available on the Sequence Read Archive (SRA) section of the National Center for Biotechnology Information (NCBI) platform BioProject PRJNA557680.

### Public data set

An online search for gene expression data obtained by RNASeq using next-generation sequencing (NGS) technology for CMC was conducted in the public GEO database (https://www.ncbi.nlm.nih.gov/geo/) [19]. The datasets GSE119810 (BioProject PRJNA489087, SRA SRP219096), GSE136197 (BioProject PRJNA561580, SRA SRP159466), and GSE135183 (BioProject PRJNA557680, SRA SRP216930) were acquired. Unlike the other RNA-Seq datasets included in this study, which used fresh frozen (FF) tissues, the GSE135183 dataset was derived from laser-capture microdissected stromal formalin-fixed and paraffin-embedded (FFPE) tissues. Raw RNASeq data were downloaded for CMC cases and their corresponding paired adjacent healthy mammary resulting in 47 cases for dataset GSE119810, 16 cases for dataset GSE136197, and 15 cases for dataset GSE135183. The quality of the raw data was evaluated using FastQC (version 0.74). The clinical characteristics available for the canines analyzed in each public dataset are listed in S2 Table.

### Primary analysis of RNASeq data

For the primary analysis of RNA-Seq data (trimming, mapping, and quantification), Trimmomatic (version 0.39) [20] was used to remove adapters and low-quality sequences. The clean reads were then aligned to the canFam4 reference genome (UU_Cfam_GSD_1.0). (https://www.ncbi.nlm.nih.gov/datasets/taxonomy/9615/) using HISAT2 (version 2.2.1) [21]. Because the libraries were prepared with the TruSeq Stranded Total RNA Sample Preparation Kit, strand-specific parameters were used during transcript quantification. Subsequently, raw gene expression counts were obtained using HTSeq-count (version 2.0.5) [22].

### Analysis of differentially expressed genes (DEGs)

Gene annotation was performed for each raw count table from the four gene expression datasets (CPA-UN, GSE119810, GSE136197, and GSE135183) obtained from the primary analysis using the corresponding GenID according to the Official Gene Symbol. Each dataset was analyzed independently to preserve its biological and technical characteristics. Subsequently, an integrative analysis was conducted to identify recurrent differentially expressed genes (DEGs) consistently observed across independent datasets. For each gene expression set, genes with no variance were filtered out, yielding a final expression matrix that was normalized and used for differential gene expression analysis between CMCs and adjacent paired healthy mammary tissues using the DESeq2 package (version 2.11.40.8) in R (version 4.2.3) [23]. Significantly DEGs were defined as those with a log2FoldChange ≥1 or ≤−1 and padj < 0.05. A Venn diagram was created to visualize the shared upregulated and downregulated DEGs among the four gene expression datasets using the Venny 2.1 tool (https://bioinfogp.cnb.csic.es/tools/venny/index2.0.2.html).

### Functional enrichment analysis

Using the Venn diagrams from the differential expression analysis, a combined list of upregulated and downregulated DEGs present in the various intersection areas across the four gene expression datasets was compiled. To preserve transparency regarding the independently generated RNASeq data produced in the present study, functional enrichment analysis was initially performed separately for the CPA-UN dataset. Subsequently, the same analysis was performed by combining CPA-UN with the publicly available GEO datasets (GSE119810, GSE136197, and GSE135183) to identify biological pathways consistently observed across independent CMC cohorts. This combined strategy enabled both dataset-specific characterization and cross-study evaluation of recurrent molecular alterations associated with CMC.

A functional enrichment analysis of this gene set was conducted in R (version 4.2.3) using the Bioconductor packages ClusterProfiler [24] to compare biological themes between gene groups and Pathview [25] to integrate and visualizing pathway-based data. The list of DEGs was used as an input set in both tools, and enrichment of Gene Ontology (GO) terms in the categories Biological Process (BP), Molecular Function (MF) and Cellular Component (CC) was evaluated, as was enrichment of metabolic pathways in the Kyoto Encyclopedia of Genes and Genomes (KEGG). The analysis was performed using the enrichGO and enrichKEGG functions from the ClusterProfiler and Pathview packages. p-values were calculated using hypergeometric tests and adjusted for multiple comparisons using the Benjamini–Hochberg method to control the false discovery rates (FDR). Terms with FDR-adjusted p-values (padj < 0.05) were considered significantly enriched. This analysis was complemented by the online platform SRplot (https://www.bioinformatics.com.cn/en) [26] and the results were graphed. The same procedure was followed for the shared downregulated DEGs, and biological terms and pathways with a padj value < 0.05 were considered statistically significant enrichments for the tumor phenotype.

Additionally, a Gene Set Enrichment Analysis (GSEA) was conducted using the raw count matrix from the CPA-UN gene expression dataset, which was normalized with the DESeq2 package (version 2.11.40.8) in R (version 4.2.3) using the median of ratios method to normalize count data and produce a ranked list of DEGs generated by DESeq2 analysis as input for GSEA (version 4.4.0) [27]. The molecular signatures affected by the DEGs were identified using the main collection H (hallmark gene sets), comprising many gene sets that represent well-defined biological states or processes from the MSigDB database (version 2026.1). The analysis compared the tumor phenotype to the control phenotype using 1000 permutations and gene set permutation mode [27–29]. Upregulated gene sets with FDR < 25% and p < 0.05 were considered statistically significant molecular signatures of the tumor phenotype. A GSEA was also performed, combining the four gene expression data sets (CPA-UN, GSE119810, GSE136197, and GSE135183) into a single matrix. Prior to integration, batch effects across datasets were corrected using the ComBat-seq method implemented in the SVA package (version 3.60.0) [30] in R (version 4.2.3) considering treating dataset origin as a batch variable to minimize technical variability and improve cross-cohort comparability. Only genes present across all datasets were retained to ensure comparability. The resulting matrix of raw counts was used as input for downstream normalization and analysis. Normalization and variance stabilization were performed using the regularized logarithm transformation (rlog) implemented in the DESeq2 package (version 2.11.40.8) in R (version 4.2.3). Size factors were estimated to account for differences in sequencing depth and library composition among samples, enabling accurate comparison of gene expression levels across the integrated datasets. The DESeq2 normalization framework models count data using a negative binomial distribution and applies internal scaling factors to correct for technical variability. Normalized expression values and differential expression statistics obtained from DESeq2 were subsequently used to construct a ranked list of DEGs as input for GSEA (version 4.4.0). The molecular signatures affected by the DEGs were identified using the same methodological approach described for the GSEA of the CPA-UN gene expression data set.

### Co-expression network analysis

As with the functional enrichment analyses, the co-expression network analysis was initially performed independently using the CPA-UN dataset to preserve transparency regarding the primary RNASeq cohort generated in the present study. Subsequently, the same analysis was performed using the merged dataset (CPA-UN, GSE 119810, GSE 136197, and GSE 135183). This approach enabled characterization of the independently generated cohort in this study and evaluation of the reproducibility of co-expression patterns across multiple datasets. Gene expression data were then normalized using the regularized logarithmic transformation (rlog) implemented in DESeq2. To construct the co-expression network, genes were ranked by expression variance across samples, and the number of genes included in the analysis was determined using the elbow method applied to the variance distribution. The Pearson correlation coefficient was calculated for all pairs of genes to assess the similarity of their expression patterns. To determine significance, a statistical test based on the p-value of the Pearson correlation coefficient was employed, with the threshold set to the minimum correlation value for padj < 0.05. Significant correlations were used to define a threshold of 0.7 and to build the co-expression network, where nodes represented genes and edges indicated significant correlations. This was done using the igraph (version 2.2.2), ggraph (version 2.2.2), tidygraph (version 1.3.1), and visNetwork (version 2.1.4) packages [31] in Bioconductor for R (version 4.2.3). Various global properties and topological metrics of the co-expression network were then calculated, and hub genes were identified within each Louvain module based on degree centrality. Differential expression results were used only for node annotation and were not involved in network construction or hub gene identification. For the merged dataset, batch effects were corrected using ComBat-seq method implemented in the SVA package (version 3.60.0) in R (version 4.2.3) before normalization and downstream analyses. Co-expression network construction and topological analyses were subsequently performed using the same methodology applied to the CPA-UN dataset.

### Deconvolution of the tumor immune infiltrate

Given that prior studies on canine tumors have used CIBERSORTx-based immune deconvolution approaches in an exploratory manner [32–34], each of the four gene expression datasets (CPA-UN, GSE119810, GSE136197, and GSE135183) was analyzed independently. This strategy was adopted to account for differences in sample composition, tissue preservation, sequencing protocols, and cohort characteristics, thereby minimizing technical heterogeneity and avoiding potential biases associated with dataset integration. This approach also enabled comparison of immune infiltration patterns across independent datasets. For each dataset, raw mRNA counts were normalized to transcripts per million mapped reads (TPM), and the relative proportions of 22 infiltrating immune cell populations were inferred using the CIBERSORTx algorithm [35]. Normalized matrices were prepared using standard gene annotation (GenID) and uploaded to the CIBERSORTx web portal (https://cibersortx.stanford.edu). The algorithm was run with the default LM22 gene signature matrix and 1000 permutations. Because LM22 was originally developed from human leukocyte transcriptional profiles, the inferred immune cell proportions in canine samples were treated as exploratory estimates rather than absolute quantifications. For each gene expression dataset, differences in the relative abundance of the 22 infiltrating immune cell types between CMC and paired adjacent healthy mammary tissue were assessed using the nonparametric Wilcoxon rank-sum test. To reduce the likelihood of false-positive findings resulting from multiple comparisons, p-values were adjusted using the Benjamini–Hochberg FDR correction. Only statistically significant differences after FDR adjustment were emphasized in the interpretation of the results. Results were visualized with box plots, which helped identify significant differences in the 22 infiltrating immune cell types between the two conditions.

### Global survival analysis and DEGs

The GSE119810 gene expression dataset was used, which included surgery date and survival status at the end of the follow-up period for the 47 CMC cases (01/08/2018). DEGs with log2FoldChange ≥1 or ≤−1 and padj < 0.05 were identified from the DESeq2 differential expression analysis. Normalization was performed using the variance-stabilizing transformation (VST) method. The normalized data were combined into a matrix along with the survival time in days and the survival status of the canines. Overall survival (OS) was defined as the time in days from diagnosis to death or last follow-up. Exploratory OS analysis was conducted for each DEG based on its median expression using the survival (version 0.5.2) and survminer (version 0.5.2) packages [31] in Bioconductor for R (version 4.2.3). Kaplan-Meier survival plots were generated, and a LogRank p < 0.05 was considered to identify DEGs with nominal associations with OS. In addition, a univariate Cox proportional hazards regression model was fitted for each gene to estimate the hazard ratio (HR) and its 95% confidence interval (CI). p-values derived from the Cox models were adjusted for multiple testing using the Benjamini–Hochberg method. Genes with significant FDR-adjusted p-values were considered associated with OS.

## Results

### Differentially expressed genes (DEGs) in canine mammary carcinoma

**Fig 1:**
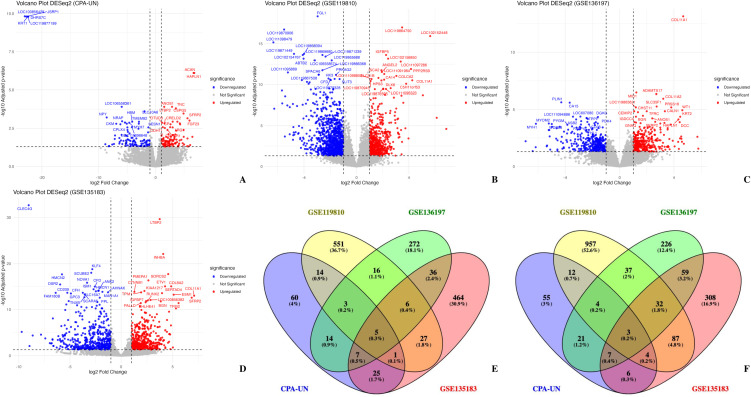
Differentially expressed genes (DEGs) in canine mammary carcinoma. The volcano plot shows upregulated and downregulated DEGs in canine CMC across the gene expression datasets CPA-UN (A), GSE119810 (B), GSE136197 (C), and GSE135183 (D). Red dots indicate upregulated DEGs, while blue dots show downregulated DEGs, selected using log2Fold Change ≥1 or ≤−1 and padj < 0.05 (the top 20 DEGs are marked). (E) The Venn diagram displays the shared upregulated DEGs among the datasets CPA-UN, GSE119810, GSE136197, and GSE135183 (*ACAN*, *COL11A1*, *EDIL3*, *NDUFA4L2* and *IGFBP5* are common to all four). (F) The Venn diagram illustrates the common downregulated DEGs across the same datasets (*TNNC1*, *PCK1*, and *METTL24* are shared in all four).

DESeq2 analysis identified 241 DEGs (129 upregulated and 112 downregulated) in the CPA-UN set (10 CMCs with their respective paired adjacent healthy mammary tissue); 1759 DEGs (623 upregulated and 1136 downregulated) in the GSE119810 set (47 CMCs with their respective paired adjacent healthy mammary tissue); 748 DEGs (359 upregulated and 389 downregulated) in the GSE136197 set (16 CMCs with their respective paired adjacent healthy mammary tissue); and 1077 DEGs (571 upregulated and 506 downregulated) in the GSE135183 set (15 CMCs with their respective paired adjacent healthy mammary tissue) (Fig 1A–1D). The list of DEGs for each gene expression dataset is shown in Supplementary S3 Table. In addition to annotated DEGs, several predicted/uncharacterized DEGs annotated with LOC identifiers were detected across the analyzed datasets. Although functional annotations were not available for these genes in the GTF file of the canFam4 reference genome, their corresponding gene biotypes were retained and included in S4 Table. A Venn diagram revealed that five upregulated genes (*ACAN*, *COL11A1*, *EDIL3*, *NDUFA4L2* and *IGFBP5*) and three downregulated genes (*TNNC1*, *PCK1*, *METTL24*) were common to all four expression datasets (Fig 1E and 1F), indicating that these eight DEGs may have stable differential expression between CMC and adjacent healthy mammary tissue.

Additionally, the Venn diagram revealed that three upregulated genes (*CSPG5MIA, COL11A2* and *MIA*) and four downregulated genes (*DES*, *MYH7*, *TNNT1* and *LMOD2*) were common to the CPA-UN, GSE119810, and GSE136197 datasets. Seven upregulated genes (*HAPLN1*, *NEFH*, *SFRP2*, *ADAMTS17*, *CCN4*, *VASH2* and *ENO1*) and seven downregulated genes (*ALDH1L1*, *IP6K3*, *ZBTB16*, *TTN*, *EEF1A2*, *SERPINB13* and *CA15*) were shared among the CPA-UN, GSE136197 and GSE135183 datasets. In addition, one upregulated gene (*SAMHD1*) and four downregulated genes (*MAN1A1*, *FGL2*, *LOC119877199* and *KRT1*) were common to the CPA-UN, GSE119810 and GSE135183 datasets. Finally, six upregulated genes (*FMOD*, *PHLDA1*, *BGN*, *INHBA*, *TNN* and *CPXM2*) and 32 downregulated genes (*DUSP1*, *NOVA1*, *PLXDC1*, *KANK3*, *KLF2*, *SELENOP*, *CCN5*, *PLIN1*, *ITIH4*, *CD34*, *CRIP1*, *PLAC9*, *CLDN5*, *EMX2*, *FABP4*, *NOX5*, *DCN*, *MGLL*, *GALNT15*, *CADM3*, *PALM*, *CFD*, *LYPD3*, *KLF4*, *CAVIN2*, *ITIH5*, *SCARA5*, *CD36*, *CLEC3B*, *MFAP5*, *PRDM8* and *GSC*) were common to the GSE119810, GSE136197 and GSE135183 datasets (Fig 1E and 1F).

### Functional enrichment in canine mammary carcinoma

To highlight functional processes potentially related to CMC, 154 upregulated DEGs and 272 downregulated DEGs were identified within from the Venn diagrams obtained in the differential expression analysis (Fig 1E and 1F), across the intersection areas of the four gene expression data sets (S5 Table). These DEGs were used to perform a functional enrichment analysis of gene ontology (GO) and Kyoto Encyclopedia of Genes and Genomes (KEGG) pathways.

**Fig 2:**
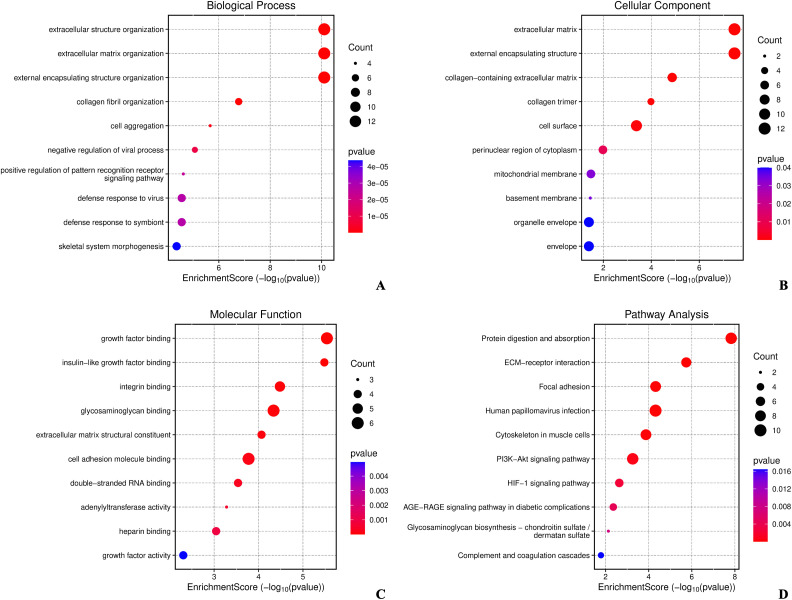
Enrichment analysis of upregulated DEGs associated with canine mammary carcinoma. From the Venn diagrams obtained in the differential expression analysis, 154 upregulated DEGs identified across the intersection areas of the four gene expression datasets were used to perform gene ontology (GO) and Kyoto Encyclopedia of Genes and Genomes (KEGG) pathway enrichment analyses. Gene ontology analysis of biological processes (A), gene ontology analysis of cellular components (B), gene ontology analysis of molecular functions (C), and KEGG pathway enrichment analysis (D) are shown. The enrichment score dot plots display the top 10 statistically significant terms for each functional biological category.

GO analysis revealed that among the upregulated DEGs in CMC included, the top 10 enriched and significant biological process (BP) terms were “extracellular matrix organization”, “extracellular structure organization”, “external encapsulating structure organization”, “collagen fibril organization”, “cell aggregation”, “negative regulation of viral process”, “positive regulation of pattern recognition receptor signaling pathway”, “defense response to virus”, “defense response to symbiont” and “skeletal system morphogenesis”. The top 10 enriched and significant cellular component (CC) terms were “external encapsulating structure”, “extracellular matrix”, “collagen-containing extracellular matrix”, “collagen trimer”, “cell surface”, “perinuclear region of cytoplasm”, “mitochondrial membrane”, “basement membrane”, “organelle envelope and envelope”. The top 10 enriched and significant molecular function (MF) terms included “growth factor binding”, “insulin-like growth factor binding”, “integrin binding”, “glycosaminoglycan binding”, “extracellular matrix structural constituent”, “cell adhesion”, “molecule binding”, “double-stranded RNA binding”, “adenylyltransferase activity”, “heparin binding” and “growth factor activity”. The top 10 enriched and significant KEGG pathways in CMC were “Protein digestion and absorption”, “ECM-receptor interaction”, “Focal adhesion”, “Human papillomavirus infection”, “Cytoskeleton in muscle cells”, “PI3K-Akt signaling pathway”, “HIF-1 signaling pathway”, “AGE-RAGE signaling pathway in diabetic complications”, “Glycosaminoglycan biosynthesis - chondroitin sulfate/ dermatan sulfate” and “Complement and coagulation cascades” (Fig 2A–2D) (S6 Table).

For downregulated DEGs, GO analysis revealed that the top 10 enriched and significant BP terms in CMC included “muscle organ development”, “striated muscle tissue development”, “muscle tissue development”, “skeletal muscle contraction”, “multicellular organismal movement”, “musculoskeletal movement”, “cardiac muscle tissue morphogenesis”, “muscle tissue morphogenesis”, “muscle structure development”, and “cardiac muscle tissue development”. The top 10 enriched and significant CC terms in CMC were “myofibril”, “contractile fiber”, “sarcomere”, “striated muscle thin filament”, “supramolecular fiber”, “supramolecular polymer”, “myofilament”, “external encapsulating structure”, “extracellular matrix” and “collagen-containing extracellular matrix”. The top 10 enriched and significant MF terms in CMC included “actin binding”, “cytoskeletal protein binding”, “collagen binding”, “calcium-dependent protein binding”, “cytokine activity”, “extracellular matrix structural constituent”, “chemokine activity”, “actin filament binding”, “monocarboxylic acid transmembrane transporter activity” and “calcium ion binding”. The top 10 enriched and significant KEGG pathways in CMC were “Cytoskeleton in muscle cells”, “Cornified envelope formation”, “Regulation of lipolysis in adipocytes”, “Fluid shear stress and atherosclerosis”, “Dilated cardiomyopathy”, “*Staphylococcus aureus* infection”, “Apelin signaling pathway”, “Motor proteins”, “Circadian entrainment”, and “Amphetamine addiction” (S1 Fig and S7 Table).

**Fig 3:**
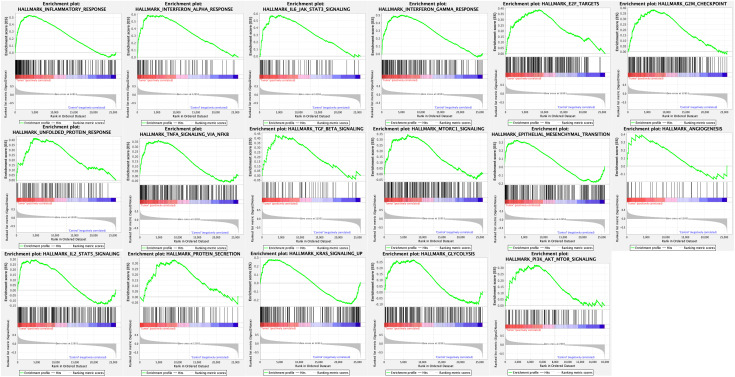
Gene Set Enrichment Analysis (GSEA) of the CPA-UN gene expression dataset. Enrichment plots are shown for upregulated gene sets from the main collection H (hallmark gene sets) with FDR < 25% and p-value < 0.05, associated with the tumor phenotype. These gene sets relate to cancer, tumorigenesis, and tumor progression.

GSEA on the CPA-UN gene expression dataset indicated that several statistically significant hallmark gene sets associated with cancer, tumorigenesis, and tumor progression were enriched in the tumor phenotype. Among these, “E2F targets”, “G2M checkpoint”, “Epithelial-mesenchymal transition”, “Angiogenesis”, “Glycolysis”, “mTORC1 signaling”, “Protein secretion”, and “TGF-β signaling” were also identified in the GSEA performed after merging the four gene expression datasets (CPA-UN, GSE119810, GSE136197, and GSE135183) into a single normalized matrix (Figs 3 and S2; S8 and S9 Tables). In contrast, the CPA-UN dataset specifically showed enrichment of statistically significant hallmark gene sets related to inflammation, immune signaling, and oncogenic signaling, including “Inflammatory response”, “IL-6–JAK–STAT3 signaling”, “TNF-α signaling via NF-κB”, “IL2–STAT5 signaling”, “Apoptosis”, “KRAS signaling up”, and “PI3K–AKT–mTOR signaling” (Fig 3 and S8 Table). Conversely, the integrated analysis uniquely identified enrichment of statistically significant hallmark gene sets “MYC targets”, “NOTCH signaling”, “Oxidative phosphorylation”, and “DNA repair”, highlighting additional biological processes consistently represented across the combined datasets (S2 Fig and S9 Table).

### Co-expression network in canine mammary carcinoma

**Fig 4:**
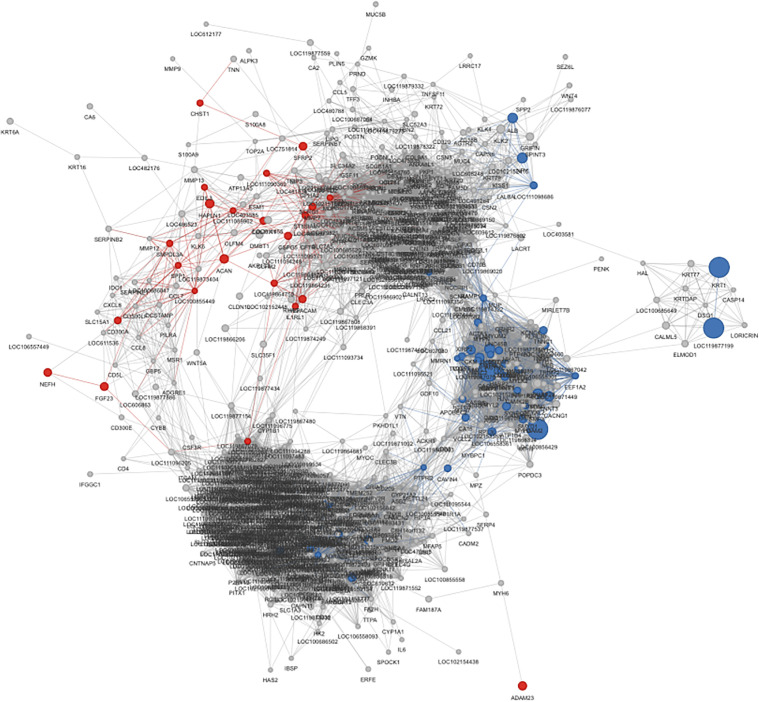
Co-expression network for the CPA-UN gene expression dataset. The network includes upregulated DEGs (red nodes), downregulated DEGs (blue nodes), and non-DEGs (gray nodes). The network was constructed using the 1000 most variable genes with an adjusted p-value (padj) < 0.05 and a Pearson correlation threshold of 0.7. Each node represents a gene, and each edge indicates the co-expression relationships established between genes based on the correlation threshold. DEGs were identified using DESeq2 and defined as genes with a log2Fold Change ≥ 1 or ≤ −1 and padj < 0.05. The size of each node reflects the gene’s log2Fold Change.

To reduce dimensionality while preserving the most informative transcriptional signals, the top 1000 most variable genes identified from the rlog-normalized expression matrix using the elbow method were selected for co-expression network construction. Pairwise Pearson correlation coefficients were computed for all selected genes, and gene pairs with correlation coefficients greater than 0.7 were retained as network edges to emphasize strong co-expression relationships and reduce weak or potentially spurious associations (Fig 4).

Following this analysis, the resulting co-expression network comprised 988 nodes and 62935 edges, indicating that most highly variable genes were incorporated into the network. Global topological analysis revealed a network density of 0.129, an average shortest path length of 2.40, and a diameter of 7.25. The network was organized into two connected components and seven modules identified through community detection analysis. Furthermore, the network exhibited a high global clustering coefficient (0.732), indicating a strongly modular organization characterized by densely interconnected groups of co-expressed genes that may represent coordinated biological processes and regulatory programs (strongly correlated genes forming clusters or functional modules). S10 Table lists the topological metrics of this co-expression network. Hub gene analysis was performed for each of the seven co-expression modules, and the top 10 genes ranked by degree were identified as module-specific hub genes (S11 Table). Module 1 was characterized by hub genes including *CALML5, LOC100685649, CASP14, DSG1, ELMOD1, KRT1, KRT77, KRTDAP, LOC119877199*, and *HAL*. Module 2 contained highly connected genes such as *MAT1A, EPCAM, FAM83F, CLDN8, ESRP1, BNIPL, RASEF, ABCC11, SHANK2*, and *PROM2*. Module 3 was represented by hub genes including *TCAP, MYLPF, CMYA5, HABP2, LMOD2, MYL1, MYOM3, TNNT3*, and *CSRP3*. Modules 4 and 5 contained the most highly connected hub genes in the network, with degree values ranging from 301 to 355, and these modules were dominated by uncharacterized LOC transcripts. Nevertheless, module 4 also included the annotated genes *KLB, CYP2A13*, and *CHRNA1*, whereas module 5 was composed almost exclusively of LOC genes among its highest-ranked hubs. Module 6 contained annotated hub genes including *ADGRE1, ESM1, MSR1, CCL8, DCSTAMP, GBP5, PILRA*, and *COL11A1* (upregulated common DEG across the CPA-UN, GSE119810, GSE136197, and GSE135183 datasets). Finally, Module 7 was composed of two hub genes, *APLN* and *VAT1L*. Additionally, co-expression modules comprising the upregulated and downregulated DEGs common to the CPA-UN, GSE119810, GSE136197, and GSE135183 datasets were identified in the co-expression network (S3 and S4 Figs). Notably, the co-expression network revealed that the upregulated DEGs ACAN, COL11A1, and EDIL3 clustered within Module 6 (S3 Fig), whereas the downregulated DEGs PCK1 and METTL24 clustered within Module 4 (S4 Fig).

For the merged gene expression dataset (CPA-UN, GSE119810, GSE136197, and GSE135183), a co-expression network was constructed from the 2000 most variable genes identified by the elbow method, using a Pearson correlation threshold of 0.7 (S5 Fig). The resulting network comprised 1907 nodes and 1501012 edges, indicating that most highly variable genes were incorporated. Global topological analysis revealed a high network density (0.826), an average shortest path length of 1.09, and a diameter of 4.55, reflecting a highly interconnected network structure. Community detection analysis identified eight modules distributed across five connected components. The network also exhibited a very high global clustering coefficient (0.951), indicating extensive local connectivity and strong co-expression among genes. The topological metrics of this co-expression network are detailed in S12 Table. Hub gene analysis was performed for each of the eight network modules, and the top 10 genes ranked by degree were selected as module-specific hub genes (S13 Table). Most of the identified hub genes corresponded to uncharacterized LOC transcripts. Among the annotated genes, module 1 contained *TMEM262* as a hub gene, module 7 included the lncRNA gene *RPPH1*, and module 8 contained the annotated genes *CSN2* and *CSN3*. In contrast, the top hub genes in modules 2, 3, 4, 5, and 6 were exclusively uncharacterized LOC transcripts (S13 Table).

### Deconvolution of the tumor immune infiltrate in canine mammary carcinoma

**Fig 5:**
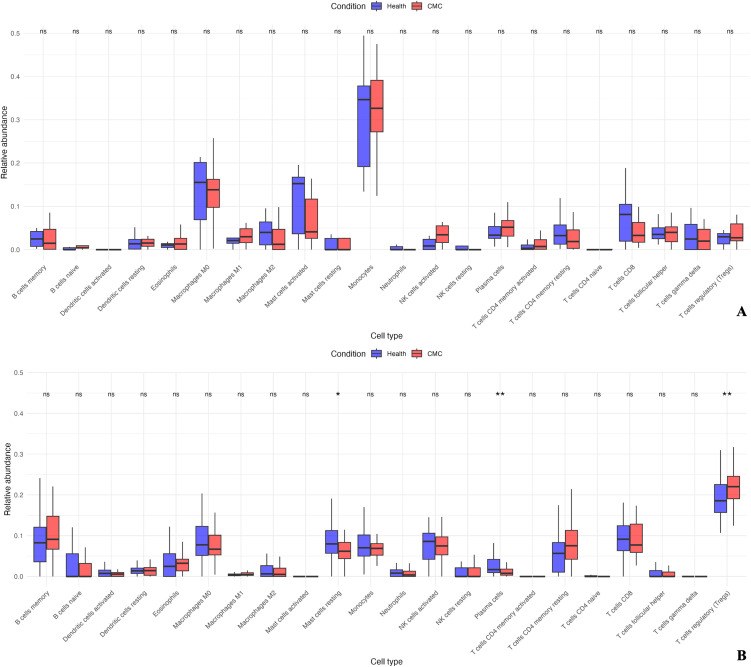
Deconvolution of tumor immune infiltrate from RNASeq. Summary of the relative proportions of 22 infiltrating immune cell types predicted from raw RNASeq counts normalized to transcripts per million (TPM) using the CIBERSORTx algorithm for the CPA-UN (A) and GSE119810 (B) gene expression datasets. Statistical comparisons were performed using the Wilcoxon signed-rank test, and the p-values displayed in the figure correspond to the Wilcoxon test results * indicates p < 0.05; ** indicates p < 0.01). p-values were additionally adjusted using the Benjamini–Hochberg false discovery rate (FDR) correction for multiple comparisons. Results that did not remain significant after FDR correction were interpreted as exploratory trends.

After normalization by converting raw mRNA counts to TPM for each gene expression dataset (CPA-UN, GSE119810, GSE136197, and GSE135183), the relative proportions of 22 infiltrating immune cell types were inferred using CIBERSORTx in an exploratory manner. For the CPA-UN gene expression dataset, no significant differences were observed in the relative abundance of the 22 infiltrating immune cell types between CMCs and paired adjacent healthy mammary tissues using the Wilcoxon signed-rank test (Fig 5A). In the GSE119810 gene expression dataset T regulatory cells (Tregs) tended to have higher median proportions in CMCs, whereas plasma cells and resting mast cells tended to show lower median proportions in CMCs compared to paired adjacent healthy tissues; however, these differences did not remain statistically significant after FDR correction for multiple comparisons (Fig 5B). Similarly, in the GSE136197 gene expression dataset, M1 macrophages tended to have higher median proportions in CMCs compared to paired adjacent healthy tissues, although this difference was not statistically significant after FDR correction (S6A Fig). In the GSE135183 gene expression dataset, activated CD4 memory T cells tended to show higher median proportions in CMCs, whereas activated dendritic cells and resting mast cells tended to have lower median proportions in CMCs compared to paired adjacent healthy tissues; however, these differences also did not remain statistically significant after FDR correction for multiple comparisons (S6B Fig). Overall, these exploratory findings should be interpreted cautiously because most observed differences did not remain statistically significant after multiple-testing correction.

### DEGs associated with overall survival in canine mammary carcinoma

The median expression of the 1759 DEGs (623 upregulated and 1136 downregulated) identified in the GSE119810 dataset (47 CMC cases with data on the surgery date and survival status at the end of the follow-up cutoff on 08/01/2018) was used for overall survival (OS) analysis of each DEG.

**Fig 6:**
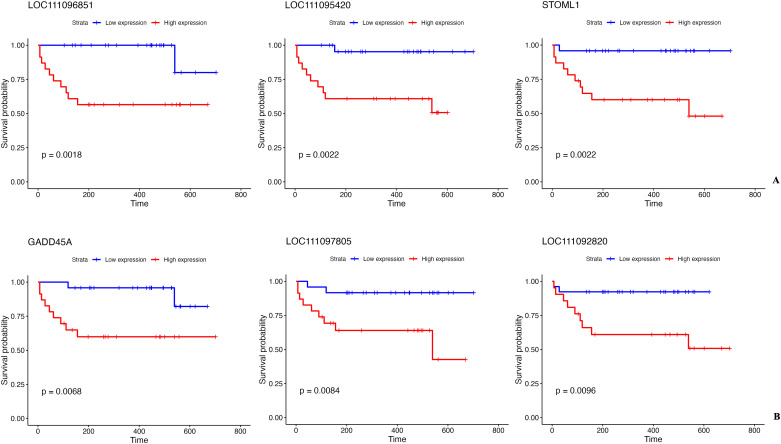
Exploratory overall survival (OS) analysis of DEGs in the GSE119810 gene expression set. Of the 1077 DEGs identified, 53 genes showed nominal associations with OS by the LogRank test (p < 0.05). Shown are the Kaplan–Meier curves for the three upregulated DEGs (A) and three downregulated DEGs (B) with the strongest nominal associations (lowest nominal LogRank p-values and highest hazard ratios). Red indicates the group of CMC dogs with higher gene expression and blue indicates the group with lower gene expression. None of the evaluated genes remained statistically significant after FDR correction in the Cox proportional hazards models; therefore, these results should be considered exploratory.

The Cox proportional hazards regression model and the Log-rank test identified 30 upregulated DEGs with nominal associations with survival (LogRank p < 0.05) (*LOC111096851, LOC111095420, STOML1, LOC480552, LOC119874434, LOC119871545, LOC111090939, SLC6A13, LOC119871897, LOC111092134, STAP1, LOC119869257, TM4SF5, LOC119873395, LOC119867511, TM4SF4, PPBP, SIDT1, NELFE, RANBP3, FAM131A, LOC111095623, DUSP26, LOC111097691, VTI1B, PPM1N, RANBP17, LOC119867810, FADS3* and *LOC102155326*) whose higher expression levels were associated with an increased risk of mortality (HR > 1). Conversely, 23 downregulated DEGs with nominal associations with survival (LogRank p < 0.05) (*GADD45A, LOC111097805, LOC111092820, FAM20A, MIR29B-2, ITGB2, DHDDS, PIGQ, MFHAS1, MBOAT1, ZCCHC17, SFN, LOC111092518, LOC119871949, LOC111095434, UBXN2A, ATP6V1FNB, LOC111092804, KRT73, LOC106558660, FYN, WASF3* and *LOC111097134*) were associated with a reduced risk of mortality (HR < 1), suggesting a potential protective effect. A substantial proportion of the DEGs showing nominal associations with OS corresponded to poorly characterized loci, particularly long non-coding RNAs (lncRNAs). Among the upregulated DEGs were *LOC111095623*, *LOC119867810*, *LOC111095420*, *LOC119871545*, *LOC119871897*, and *LOC111096851*, whereas several downregulated DEGs, including *LOC106558660*, *LOC111095434*, *LOC119871949*, *LOC111097134*, *LOC111097805*, *LOC111092518*, *LOC111092820*, and *LOC111092804*, were also annotated as lncRNAs. However, the 95% IC and multiple testing correction using the Benjamini–Hochberg method, showed that none of the genes remained statistically significant (FDR > 0.05) (S14 Table), indicating that these findings should be interpreted as exploratory and require validation in larger cohorts. Kaplan–Meier survival curves for the three upregulated and three downregulated DEGs exhibiting the strongest nominal associations with overall survival (OS) and the highest hazard ratios (HRs) are shown in Fig 6.

## Discussion

The alternative canine biomodel for HBC research offers a valuable research opportunity because CMC occurs spontaneously in dogs of all ages and shares features with HBC, including epidemiology, age at onset, hormonal causes, clinical progression, histopathological similarities, mutation profiles, gene expression changes, and factors influencing clinical outcomes [11,12,36–38]. The present integrative transcriptomic analysis identified recurrent transcriptional alterations associated with CMC across four independent RNA-seq datasets (CPA-UN, GSE119810, GSE136197, and GSE135183), providing insights into biological processes potentially involved in tumor development and progression. Although each dataset exhibited substantial variability in the number of DEGs, reflecting differences in cohort size, biological heterogeneity, and residual technical variation across studies, the integrative approach identified a consistent core of transcriptional changes. Across all data sets, the integrative analysis identified eight genes (*ACAN*, *COL11A1*, *EDIL3*, *NDUFA4L2*, *IGFBP5*, *TNNC1*, *PCK1*, and *METTL24*) that showed consistent differential expression. Although only a limited number of DEGs were shared across all datasets, the recurrent identification of these genes across independent cohorts suggests that they may represent robust, common transcriptional programs potentially associated with CMC development and progression. This also highlights differences in transcriptional profiles between CMC and adjacent healthy mammary tissue. At the same time, the marked variability across datasets underscores the biological and technical challenges inherent in transcriptomic studies of CMC. Although batch correction was performed before downstream analyses, residual technical variability arising from differences in sample collection, tissue processing, preservation methods, sequencing protocols, and cohort composition may still have contributed to the observed heterogeneity. Differences in tissue sampling procedures and tissue composition may influence gene expression profiles. Variations in the proportions of epithelial, stromal, inflammatory, or adjacent non-neoplastic tissues across the analyzed samples, together with differences in tissue preservation methods, may partially explain discrepancies among datasets. Therefore, these findings should be interpreted cautiously and underscore the need for further validation in larger and more standardized cohorts. The limited overlap across datasets further underscores the challenges of identifying reproducible transcriptomic signatures in CMC. Differences in tissue sampling, preservation methods, sequencing protocols, cohort composition, and analytical workflows may all contribute to the observed variability. These findings emphasize the need for standardized methodologies and larger multicenter studies to improve the reproducibility and robustness of transcriptomic research in CMC. Previous research has shown similar expression patterns for these eight DEGs in HBC and other human cancers, supporting their potential biological relevance to CMC progression and aggressiveness. Notably, these genes have been implicated in ECM remodeling, tumor progression, metabolic adaptation, and TME interactions in human cancers, reinforcing their potential biological relevance in CMC.

Among the recurrently upregulated genes identified across datasets, *EDIL3* (EGF-Like Repeats and Discoidin Domains 3), *COL11A1* (Collagen Type XI Alpha 1 Chain), *ACAN* (Aggrecan/Versican Proteoglycan Family), and *IGFBP5* (Insulin-like Growth Factor-Binding Protein 5) are functionally linked to extracellular matrix (ECM) organization and remodeling, cell adhesion, and interactions with the tumor microenvironment (TME). Previous studies in human cancers have linked these genes to processes including angiogenesis, epithelial–mesenchymal transition (EMT), invasion, tumor progression, and, in some cases, unfavorable clinical outcomes [39–53]. *NDUFA4L2* (NADH Dehydrogenase [Ubiquinone] 1 Alpha Subcomplex, 4-Like 2) and *PCK1* (Phosphoenolpyruvate Carboxykinase 1) are involved in metabolic regulation and cellular adaptation to TME stress. *NDUFA4L2* participates in mitochondrial respiration and oxidative stress responses and is often linked to tumor stem cell development, microsatellite instability, tumor mutational burden, immune cell infiltration, reactive oxygen species (ROS) production, disease progression, and poor OS across many types of human cancer [54–59]. *PCK1* (Phosphoenolpyruvate Carboxykinase 1) plays a central role in metabolic reprogramming and has been associated with tumor progression, epigenetic changes, TME remodeling and worse prognosis in several human cancer types [60–81]. In contrast, the recurrent downregulation of *TNNC1* (Troponin C1) and *METTL24* (Methyltransferase Like 24) may reflect the loss of normal mammary tissue structural and regulatory functions during tumor development. *TNNC1* is associated with contractile and cytoskeletal processes, and its role in cancer appears to be tumor type-dependent. In human cancers, *TNNC1* has been linked to EMT, invasion, and metastatic potential [82–86]. Likewise, *METTL24* belongs to a family of proteins involved in cellular regulatory processes and has been associated with poor prognosis in several human cancers [87–89]. In addition, some of these genes have been investigated as potential diagnostic, prognostic, or therapeutic biomarkers in specific human tumor types, although their clinical utility remains context-dependent and requires further validation [44,45,89–99]. Although their specific roles in CMC remain to be experimentally validated, their recurrent differential expression aligns with enrichment analyses and suggests that these genes may serve as candidate molecular markers for CMC development and progression, warranting further investigation in independent cohorts and functional studies. In addition to the eight DEGs shared across all four datasets, several genes were consistently identified in three-dataset comparisons, including *MIA, COL11A2, HAPLN1, SFRP2, CCN4, FMOD, BGN,* and *INHBA* among the upregulated genes, and *DES, TNNT1, LMOD2, KRT1, KLF4, CD36,* and *DCN* among the downregulated genes. Many of these genes have been associated with human cancers, including HBC, as well as with ECM remodeling, cell differentiation, and tumor progression, supporting their potential relevance to CMC biology [100–113].

From an integrative perspective, GO and KEGG enrichment analyses of upregulated DEGs revealed that genes with higher transcript levels in CMC were predominantly associated with ECM remodeling, cell adhesion, and TME interactions. Enrichment of BP terms, together with CC terms related to the ECM and basement membrane, suggests extensive stromal remodeling and structural reorganization within the TME. Consistent with this, enriched MF categories further support the involvement of ECM-mediated signaling and cell–ECM interactions in CMC progression. In addition, several significantly enriched pathways, including ECM–receptor interaction, focal adhesion, and the PI3K–Akt signaling pathway, are associated with cell survival, proliferation, migration, and invasive behavior, highlighting molecular mechanisms commonly linked to tumor progression and metastasis. The enrichment of immune- and stress-related processes further suggests the presence of inflammatory and immune responses within the TME. Collectively, these findings indicate that the transcriptomic alterations observed in CMC are characterized not only by dysregulation of structural ECM components but also by the activation of signaling pathways involved in tumor progression, immune modulation, and cellular adaptation. For downregulated DEGs, GO enrichment analysis revealed a predominance of BP terms associated with muscle development, contractile function, and tissue organization. Similarly, enriched CC terms indicate reduced expression of genes involved in cytoskeletal integrity and contractile machinery. MF categories further support the suppression of structural and contractile programs in CMC. These findings may reflect the progressive loss of normal mammary gland architecture and differentiation during CMC development and progression. In particular, the downregulation of muscle- and contractility-related genes could be associated with disruption of myoepithelial cell function and cytoskeletal remodeling, processes frequently linked to tumor invasion and loss of tissue integrity. Consistent with this, enriched KEGG pathways, such as “cytoskeleton in muscle cells” and “apelin signaling pathway”, suggest alterations in cellular mechanics, adhesion, and tissue homeostasis within the CMC TME. Collectively, these results suggest that CMC progression is characterized not only by the activation of tumor-promoting signaling pathways, ECM remodeling, and proliferative processes, but also by the loss of structural and differentiation programs characteristic of adjacent healthy canine mammary tissue. All of these pathways are involved in signaling processes related to proliferation, survival, and metastasis within the HBC TME [114,115].

GSEA consistently identified enrichment of hallmark gene sets associated with tumor progression, proliferation, extracellular matrix remodeling, immune signaling, and metabolic adaptation in the tumor phenotype. Across analyses, recurrent enrichment of pathways such as “E2F targets”, “G2M checkpoint”, “MYC targets”, and “DNA repair” suggested increased proliferative activity and cell-cycle dysregulation in CMC. Similarly, enrichment of “mTORC1 signaling”, “PI3K-AKT-mTOR signaling”, “KRAS signaling up”, and “TGF-β signaling” indicated activation of molecular pathways commonly involved in tumor growth, survival, and invasive behavior. Gene sets related to TME interactions and inflammatory responses, including “Inflammatory response”, “TNF-α signaling via NF-κB”, “IL6-JAK-STAT3 signaling”, “IL2-STAT5 signaling”, “Angiogenesis”, and “Epithelial–mesenchymal transition”, further supported the presence of immune modulation, stromal remodeling, and pro-metastatic transcriptional programs in CMC. In addition, enrichment of “Glycolysis”, “Oxidative phosphorylation”, and “Protein secretion” suggested metabolic and secretory adaptations that may contribute to tumor progression. Overall, the consistent enrichment of these biological themes across independent and integrated analyses suggests coordinated transcriptional patterns that may be related to proliferation, immune regulation, metabolic reprogramming, and TME remodeling in CMC. However, these findings should be interpreted as enrichment-based transcriptomic signatures rather than direct evidence of pathway activation. Given the variability among datasets and the potential influence of residual technical heterogeneity, the identified pathways should be considered putative biological processes requiring further functional validation.

Co-expression network analysis revealed a highly organized transcriptional architecture in the CPA-UN dataset, marked by strong modularity and extensive local connectivity. The high clustering coefficient and the presence of seven co-expression modules suggest that genes do not act independently but rather as coordinated functional units, reflecting underlying biological programs associated with CMC. The biological relevance of the identified modules is supported by the functional characteristics of their hub genes. Module 1 was characterized by genes involved in epithelial differentiation (*KRT1, KRT77, DSG1,* and *CASP14*), whereas module 2 contained epithelial-associated genes such as *EPCAM, CLDN8,* and *ESRP1*, supporting the relevance of epithelial regulatory networks in tumor biology. Module 3 was enriched for muscle-related genes (*MYLPF, MYL1, TNNT3,* and *MYOM3*), potentially reflecting stromal or myoepithelial components within the TME. In contrast, modules 4 and 5 were dominated by highly connected uncharacterized LOC transcripts. Interestingly, module 4 also included annotated genes such as *KLB*, *CYP2A13*, and *CHRNA1*, suggesting that these modules may represent poorly characterized biological processes that warrant further investigation. The predominance of LOC transcripts among highly connected nodes highlights the limited functional annotation currently available for the canine genome and suggests that additional studies may uncover novel regulators of CMC biology. Notably, module 6 contained hub genes- related to immune- and stromal functions, including *ADGRE1, MSR1, CCL8,* and *COL11A1*, supporting the role of inflammatory and ECM remodeling processes in tumor progression. Similarly, the merged-dataset network showed strong connectivity and was dominated by LOC transcripts, indicating that poorly characterized genes may represent an important yet largely unexplored component of the molecular architecture of CMC. Among the annotated genes were *TMEM262*, *RPPH1*, *CSN2,* and *CSN3*. This finding underscores the need for improved annotation of the canine transcriptome and suggests that currently uncharacterized genes may contribute substantially to the molecular architecture of CMC. Many of these genes have previously been implicated in different human cancer types, including HBC, suggesting potential relevance in CMC [116–132]

Immune deconvolution analysis using CIBERSORTx did not identify statistically significant differences in immune cell populations between CMCs and paired adjacent healthy mammary tissues after FDR correction for multiple comparisons. Although several immune cell populations, including regulatory T cells (Tregs), M1 macrophages, activated CD4 memory T cells, plasma cells, and resting mast cells, showed trends toward differential abundance in some datasets, these findings should be considered exploratory and require validation in larger cohorts. Nevertheless, some recurrent trends were observed across the independent datasets. In particular, increased proportions of Tregs, M1 macrophages, activated CD4 memory T cells, and resting NK cells, together with reduced proportions of plasma cells, resting mast cells, and CD8 + T cells, were detected in CMCs compared with paired adjacent healthy mammary tissues across one or more datasets. It is important to note that CMCs showed a tendency toward infiltration by protumoral and immunosuppressive cells compared with matched adjacent healthy tissues, which could contribute to tumor progression and a poorer prognosis [133]. Although these findings should be interpreted cautiously because they are not statistically significant after multiple-testing correction, they may suggest the presence of immune-regulatory and inflammatory processes within the CMC TME. An important observation was the variability in immune-cell composition across datasets. This variability may reflect biological differences among tumors but could also be influenced by technical factors, including sample collection, tissue preservation, sequencing protocols, and cohort composition. Given these limitations and the indirect nature of transcriptome-based immune deconvolution, the inferred immune infiltration patterns should be treated as exploratory. CIBERSORTx relies on the LM22 reference signature matrix, originally derived from human immune cell populations, and has not been validated for canine tissues. Consequently, species-specific differences in immune cell transcriptional profiles may affect the accuracy of cell proportion estimates and should be considered when interpreting these results. Nevertheless, the partially recurrent trends observed across independent cohorts support further investigation of the immune TME in CMC using larger datasets and complementary experimental approaches.

The exploratory survival analysis identified 53 DEGs with nominal associations with overall survival (OS) in the GSE119810 cohort, comprising 30 upregulated and 23 downregulated genes. Among the upregulated DEGs, the genes with the strongest nominal associations were *TM4SF5, TM4SF4, PPBP, STAP1*, and *DUSP26.* Among the downregulated DEGs, the genes with the strongest nominal associations were *GADD45A, ITGB2, FYN, WASF3*, and *SFN*. However, these findings should be interpreted with caution. Importantly, although several genes showed nominal significance in Kaplan–Meier analyses, none remained statistically significant after multiple-testing correction. Therefore, these results should be considered exploratory and hypothesis-generating rather than evidence for validated prognostic biomarkers. The discrepancy between nominal and FDR-adjusted significance underscores the challenges of identifying robust prognostic markers from transcriptomic datasets with limited sample sizes. Notably, several candidate genes identified in the survival analysis are involved in pathways related to cell proliferation, immune regulation, and TME interactions. These biological themes were also recurrently observed in the enrichment analyses, indicating convergence across the analytical approaches used in this study. Many of these genes have been implicated in OS across multiple human cancer types, suggesting potential prognostic relevance in CMC [134–167]. Another observation was that a substantial proportion of genes showing nominal associations with OS mapped to poorly characterized loci, particularly long non-coding RNAs (lncRNAs). Although the biological functions of most of these transcripts remain unknown, growing evidence indicates that lncRNAs can regulate tumor proliferation, invasion, metastasis, and immune responses [168]. Therefore, these findings may highlight previously underexplored non-coding transcriptional components of CMC biology that warrant further investigation. Nevertheless, given the limited sample size (n = 47) and the absence of statistically significant associations after multiple-testing correction, these findings should be considered exploratory and hypothesis-generating. Therefore, the identified genes represent candidate prognostic markers that require validation in larger independent cohorts and further functional characterization before any conclusions regarding their prognostic or clinical relevance can be drawn.

Although this study provides novel insights into the transcriptomic landscape of CMC, several limitations should be acknowledged. The gene expression datasets were derived from relatively small and heterogeneous cohorts, and the associated clinical information was limited and not fully standardized, potentially introducing bias and reducing the robustness of some associations. Differences in tissue sampling, preservation and sequencing protocols, and cohort composition across studies likely contributed to the substantial variability observed between datasets. The marked inter-dataset heterogeneity and limited overlap of DEGs further underscore the challenges of identifying reproducible transcriptomic signatures in CMC and highlight the need for standardized experimental and analytical approaches in future studies. In addition, the lack of experimental validation precludes definitive conclusions regarding the functional and clinical relevance of the identified genes. Therefore, the findings should be interpreted as exploratory and hypothesis-generating. Further studies in larger, well-characterized cohorts, together with functional validation, are required to confirm the relevance of the proposed candidate molecular markers and to establish a clearer link between molecular alterations and diagnostic or prognostic outcomes in CMC.

## Conclusions and future perspectives

In conclusion, through integrated bioinformatics analysis of gene expression datasets, including an independently generated dataset from the present study and publicly available RNASeq datasets. Using NGS technology, we identified a set of candidate genes with consistent differential expression patterns in CMC. Five genes (*ACAN*, *COL11A1*, *EDIL3*, *NDUFA4L2*, and *IGFBP5*) were consistently upregulated, while three genes (*TNNC1*, *PCK1*, and *METTL24*) were consistently downregulated in CMC samples compared with healthy mammary gland controls. Notably, similar expression patterns for several of these candidate biomarkers have been reported in HBC studies, suggesting potential cross-species relevance; however, these observations remain indirect and require further validation. Functional enrichment analysis indicated that these candidate genes are associated with biological processes related to tumor-associated pathways; however, these results should be interpreted as transcriptomic associations rather than evidence of functional activity. Deconvolution of tumor immune infiltrates from RNASeq data revealed heterogeneity in CMC immune composition across datasets, but these findings were exploratory and limited by the absence of statistical significance after correction for multiple testing. Survival analysis identified 53 additional candidate biomarkers (30 DEGs upregulated and 23 DEGs downregulated) associated with OS in a single dataset; however, these associations did not remain significant after multiple-testing correction and should therefore be considered hypothesis-generating.

Overall, the present study provides a preliminary transcriptomic framework for CMC that requires further validation and functional investigation. The findings provide a basis for experimental validation of the proposed candidate genes, which should be tested in larger, better-characterized cohorts with standardized clinical annotation to clarify their roles in tumorigenesis, progression, and tumor aggressiveness. Future research should prioritize longitudinal study designs and the integration of multi-omics data, including transcriptomic, genomic, epigenomic, and proteomic approaches, to refine and strengthen molecular signatures for diagnosis, risk assessment, and prognosis. In addition, computational strategies, including machine learning-based models, may help identify of clinically relevant patterns; however, these approaches require robust external validation to ensure generalizability and clinical applicability. Finally, systematic comparative studies among CMC, HBC, and other oncological models will be essential to better define cross-species similarities and differences, thereby supporting the translational relevance of canine models within comparative oncology.

## Supporting information

S1 Fig Enrichment analysis of downregulated DEGs related to canine mammary carcinoma. From the Venn diagrams obtained in the differential expression analysis, 272 downregulated DEGs identified across the intersection areas of the four gene expression datasets were used to perform gene ontology (GO) and Kyoto Encyclopedia of Genes and Genomes (KEGG) pathway enrichment analyses. Gene ontology analysis of biological processes (A), gene ontology analysis of cellular components (B), gene ontology analysis of molecular functions (C), and KEGG pathway enrichment analysis (D). The top 10 terms for each statistically significant functional biological category are displayed in the enrichment score dot plot.
(TIFF)

S2 Fig Gene Set Enrichment Analysis (GSEA) of the combined gene expression datasets CPA-UN, GSE119810, GSE136197, and GSE135183. Enrichment plots are shown for upregulated gene sets from the main collection H (hallmark gene sets) with FDR < 25% and p-value < 0.05 associated with the tumor phenotype. These gene sets relate to cancer, tumorigenesis, and tumor progression.
(TIFF)

S3 Fig Module in the co-expression network of the CPA-UN gene expression dataset for the upregulated DEGs common across the CPA-UN, GSE119810, GSE136197, and GSE135183 datasets. The co-expression module obtained with an adjusted p-value (padj) < 0.05 and a Pearson correlation threshold of 0.7 is shown for *ACAN*, *COL11A1*, and *EDIL3*. Each node represents a gene, and each edge indicates the co-expression relationships between genes based on the correlation threshold. DEGs were identified using DESeq2 and defined as genes with a log2Fold Change ≥ 1 or ≤ −1 and padj < 0.05. The size of each node reflects the log2Fold Change of the corresponding gene.
(TIFF)

S4 Fig Modules in the co-expression network of the CPA-UN gene expression dataset for the downregulated DEGs common across the CPA-UN, GSE119810, GSE136197, and GSE135183 datasets. The co-expression module obtained with an adjusted p-value (padj) < 0.05 and a Pearson correlation threshold of 0.7 is shown for TNNC1 (A), and for *PCK1* and *METTL24* (B). Each node represents a gene, and each edge indicates the co-expression relationships established between genes based on the correlation threshold. DEGs were identified using DESeq2 and defined as genes with a log2Fold Change ≥ 1 or ≤ −1 and padj < 0.05. The size of each node reflects the log2Fold Change of the corresponding gene.
(TIFF)

S5 Fig Co-expression network for the combined gene expression datasets CPA-UN, GSE119810, GSE136197, and GSE135183. The network includes upregulated DEGs (red nodes), downregulated DEGs (blue nodes), and non-DEGs (gray nodes). The network was constructed using the 2000 most variable genes with an adjusted p-value (padj) < 0.05 and a Pearson correlation threshold of 0.7. Each node represents a gene, and each edge indicates the co-expression relationships between genes based on the correlation threshold. DEGs were identified using DESeq2 and defined as genes with a log2Fold Change ≥ 1 or ≤ −1 and padj < 0.05. The size of each node reflects the gene’s log2FoldChange.
(TIFF)

S6 Fig Deconvolution of tumor immune infiltrates from RNASeq. Summary of the relative proportions of 22 infiltrating immune cell types predicted from raw RNASeq counts normalized to transcripts per million (TPM) using the CIBERSORTx algorithm for the gene expression datasets GSE136197 (A) and GSE135183 (B). Statistical comparisons were performed using the Wilcoxon signed-rank test, and the p-values displayed in the figure correspond to the Wilcoxon test results (* indicates p < 0.05; ** indicates p < 0.01). p-values were additionally adjusted using the Benjamini–Hochberg false discovery rate (FDR) correction for multiple comparisons. Results that were not significant after FDR correction were interpreted as exploratory trends.
(TIFF)

S1 Table Summary of the RNA sequencing data statistics for CPA-UN. (XLSX)

S2 Table Clinical characteristics of canines with mammary carcinoma from the public datasets GSE119810, GSE136197 and GSE135183 included in the study. (XLSX)

S3 Table Differentially expressed genes (DEGs) in the CPA-UN, GSE119810, GSE136197, and GSE135183 datasets. (XLSX)

S4 Table Predicted or uncharacterized DEGs annotated with LOC identifiers detected across the CPA-UN, GSE119810, GSE136197, and GSE135183 datasets. (XLSX)

S5 Table Differentially expressed genes (DEGs) shared across CPA-UN, GSE119810, GSE136197, and GSE135183 datasets. (XLSX)

S6 Table Functional enrichment analysis of gene ontology and Kyoto Encyclopedia of Genes and Genomes (KEGG) pathways for upregulated differentially expressed genes (DEGs) shared across the CPA-UN, GSE119810, GSE136197, and GSE135183 datasets. (XLSX)

S7 Table Functional enrichment analysis of gene ontology and Kyoto Encyclopedia of Genes and Genomes (KEGG) pathways for downregulated differentially expressed genes (DEGs) shared across the CPA-UN, GSE119810, GSE136197, and GSE135183 datasets. (XLSX)

S8 Table Gene Set Enrichment Analysis (GSEA) for canine mammary carcinoma cases in the CPA-UN dataset. (XLSX)

S9 Table Gene Set Enrichment Analysis (GSEA) for canine mammary carcinoma cases in the merged dataset (CPA-UN, GSE119810, GSE136197, and GSE135183). (XLSX)

S10 Table Topological metrics of the co-expression network for the 1000 most variable genes with an adjusted p-value (padj) < 0.05 and a Pearson correlation threshold of 0.7 in the CPA-UN dataset. (XLSX)

S11 Table Topological metrics of the co-expression network for the 2000 most variable genes with an adjusted p-value (padj) < 0.05 and a Pearson correlation threshold of 0.7 in the merged dataset (CPA-UN, GSE119810, GSE136197, and GSE135183). (XLSX)

S12 Table Hub genes of the co-expression network for the 1000 most variable genes with an adjusted p-value (padj) < 0.05 and a Pearson correlation threshold of 0.7 in the CPA-UN dataset. (XLSX)

S13 Table Hub genes of the co-expression network for the 1000 most variable genes with an adjusted p-value (padj) < 0.05 and a Pearson correlation threshold of 0.7 in the merged dataset (CPA-UN, GSE119810, GSE136197, and GSE135183). (XLSX)

S14 Table Overall survival analysis of the GSE119810 dataset. (XLSX)
