# Supplementary figures and images for: Diagnostic and prognostic values of differentially expressed genes in canine mammary carcinoma: An integrated bioinformatics analysis
Source: PLoS One. 2026 Jul 20;21(7):e0354033. doi: 10.1371/journal.pone.0354033 (PMC13384312; doi:10.1371/journal.pone.0354033)

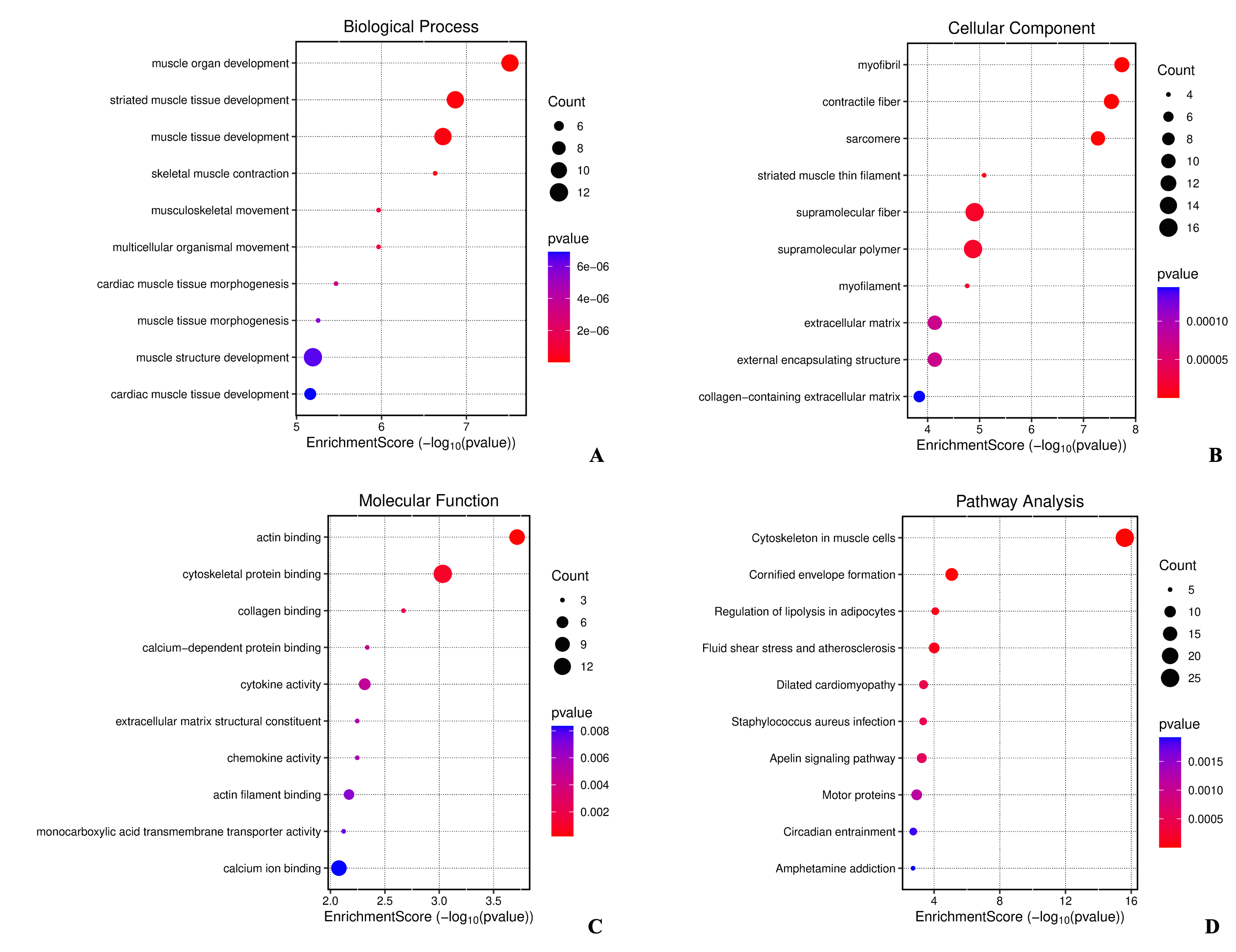

Supplement: S1 Fig — From the Venn diagrams obtained in the differential expression analysis, 272 downregulated DEGs identified across the intersection areas of the four gene expression datasets were used to perform gene ontology (GO) and Kyoto Encyclopedia of Genes and Genomes (KEGG) pathway enrichment analyses. Gene ontology analysis of biological processes (A), gene ontology analysis of cellular components (B), gene ontology analysis of molecular functions (C), and KEGG pathway enrichment analysis (D). The top 10 terms for each statistically significant functional biological category are displayed in the enrichment score dot plot. (TIFF) [file pone.0354033.s001.tiff]

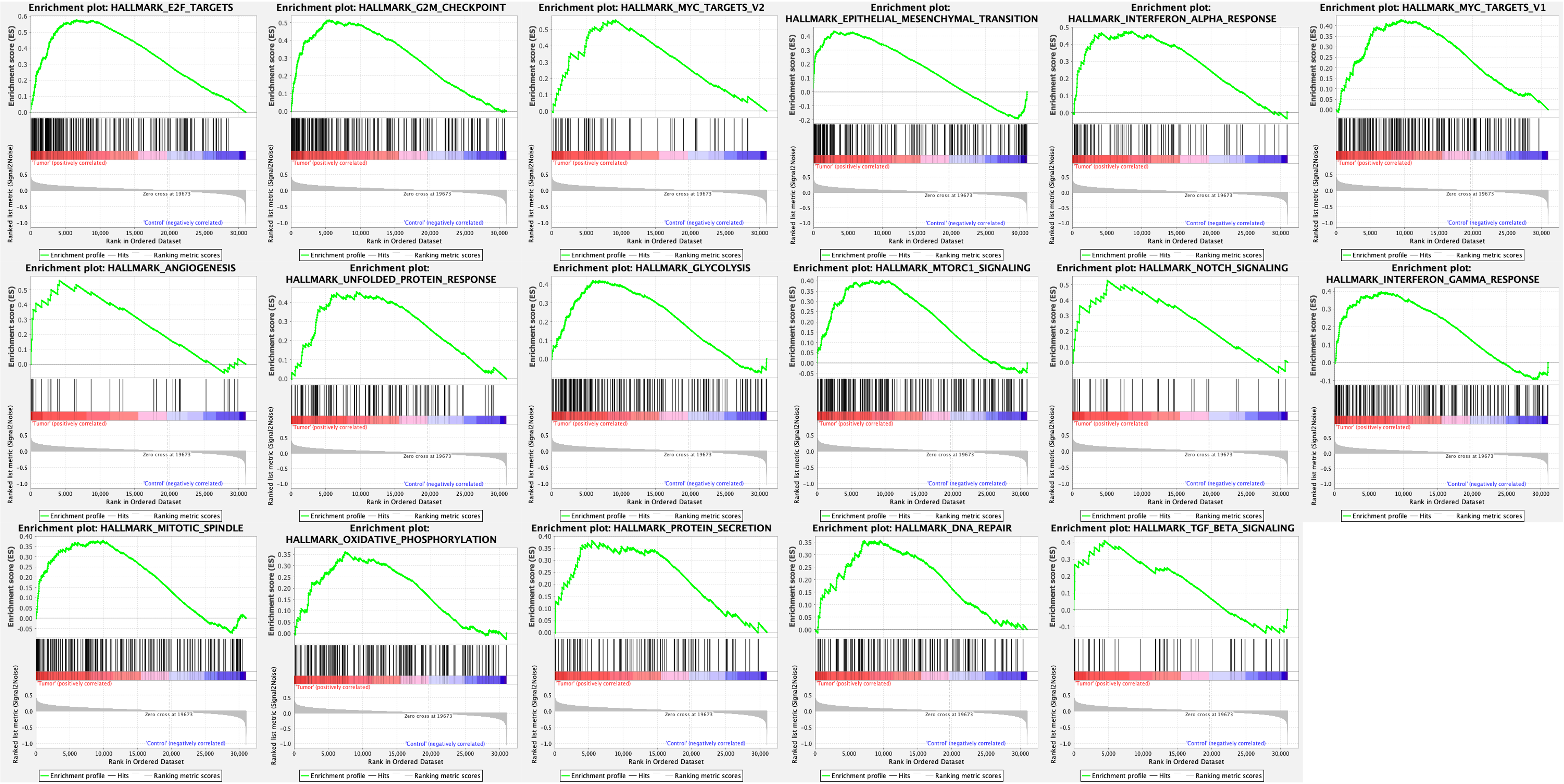

Supplement: S2 Fig — Enrichment plots are shown for upregulated gene sets from the main collection H (hallmark gene sets) with FDR < 25% and p-value < 0.05 associated with the tumor phenotype. These gene sets relate to cancer, tumorigenesis, and tumor progression. (TIFF) [file pone.0354033.s002.tiff]

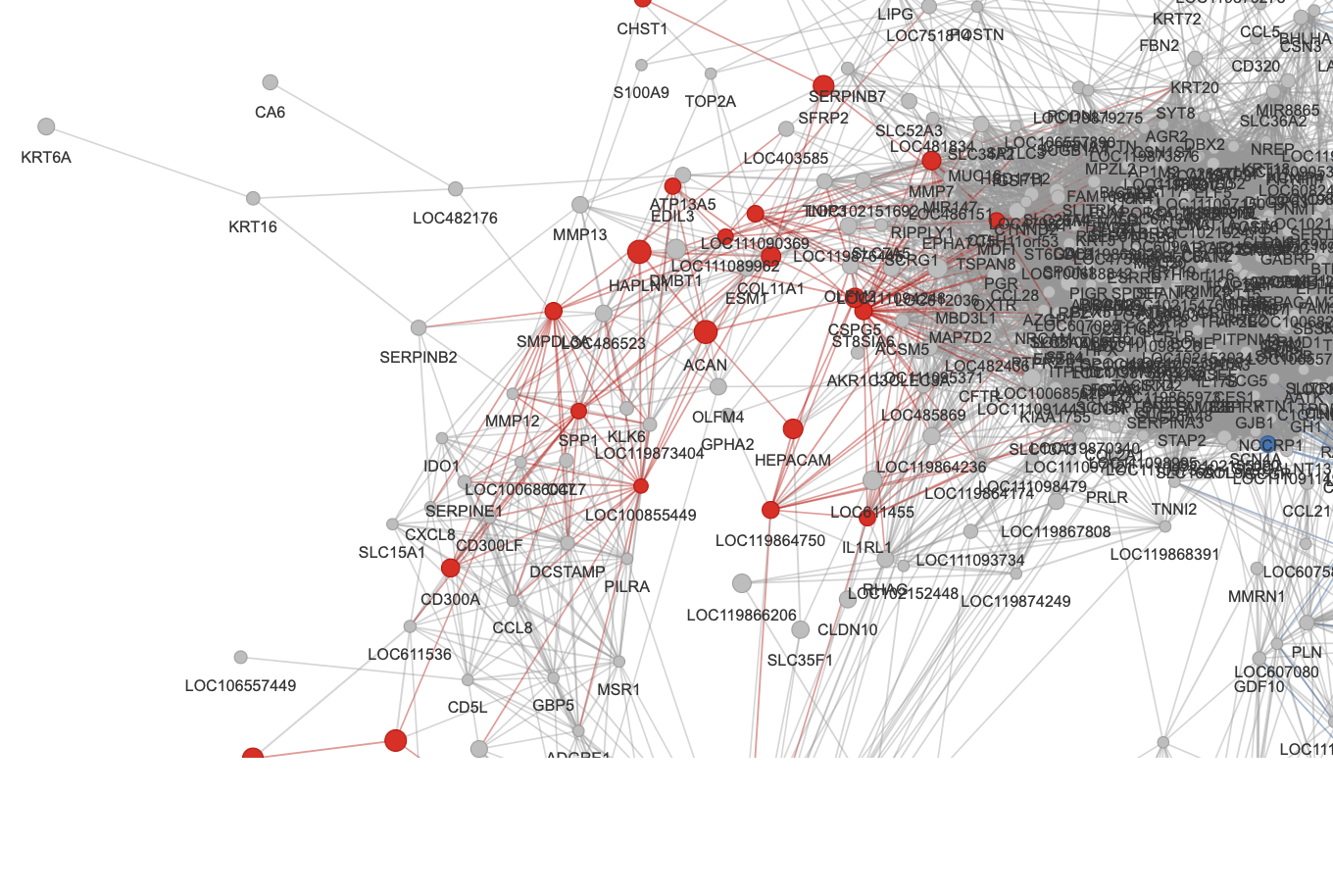

Supplement: S3 Fig — The co-expression module obtained with an adjusted p-value (padj) < 0.05 and a Pearson correlation threshold of 0.7 is shown for ACAN, COL11A1, and EDIL3. Each node represents a gene, and each edge indicates the co-expression relationships between genes based on the correlation threshold. DEGs were identified using DESeq2 and defined as genes with a log2Fold Change ≥ 1 or ≤ −1 and padj < 0.05. The size of each node reflects the log2Fold Change of the corresponding gene. (TIFF) [file pone.0354033.s003.tiff]

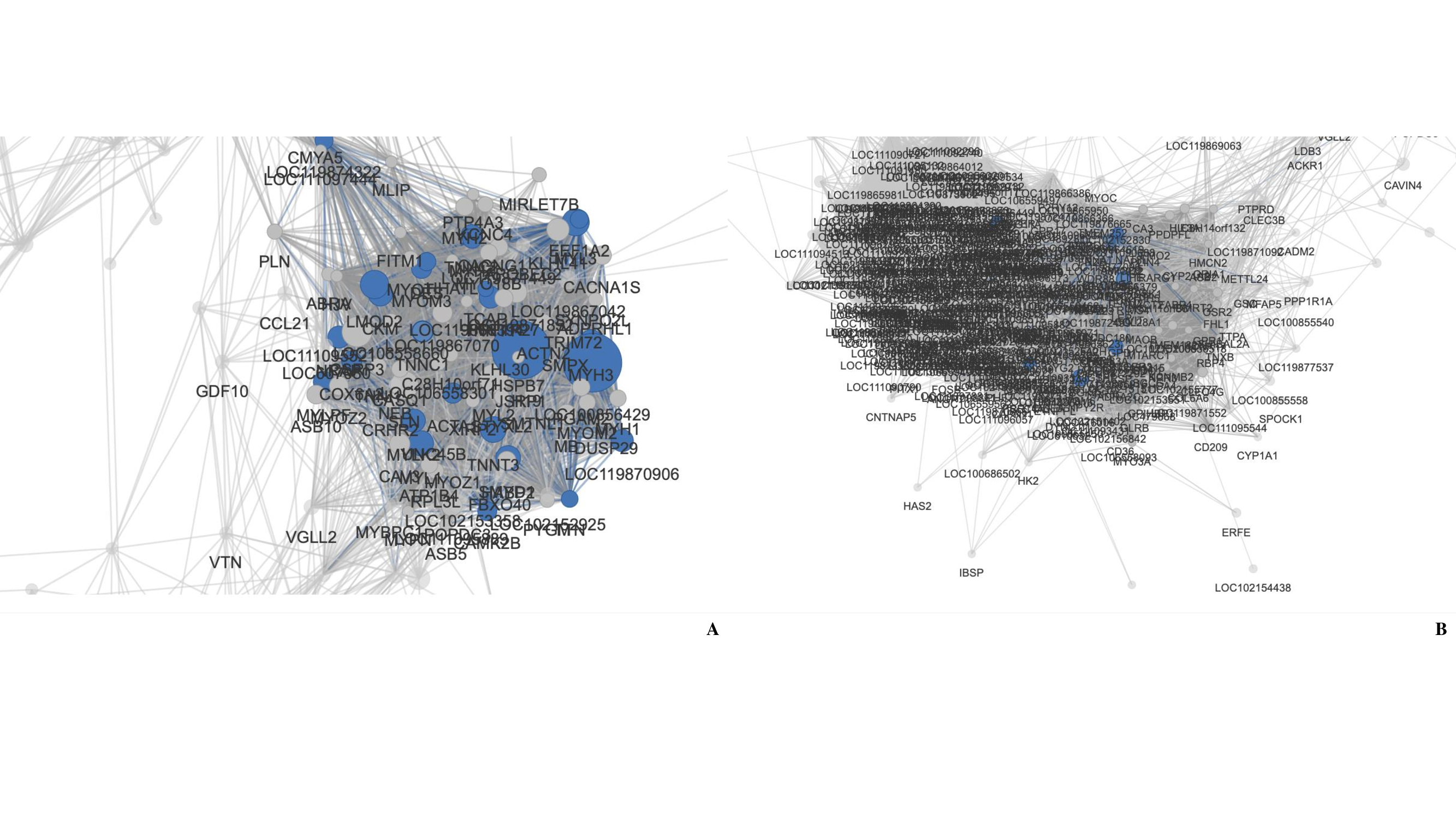

Supplement: S4 Fig — The co-expression module obtained with an adjusted p-value (padj) < 0.05 and a Pearson correlation threshold of 0.7 is shown for TNNC1 (A), and for PCK1 and METTL24 (B). Each node represents a gene, and each edge indicates the co-expression relationships established between genes based on the correlation threshold. DEGs were identified using DESeq2 and defined as genes with a log2Fold Change ≥ 1 or ≤ −1 and padj < 0.05. The size of each node reflects the log2Fold Change of the corresponding gene. (TIFF) [file pone.0354033.s004.tiff]

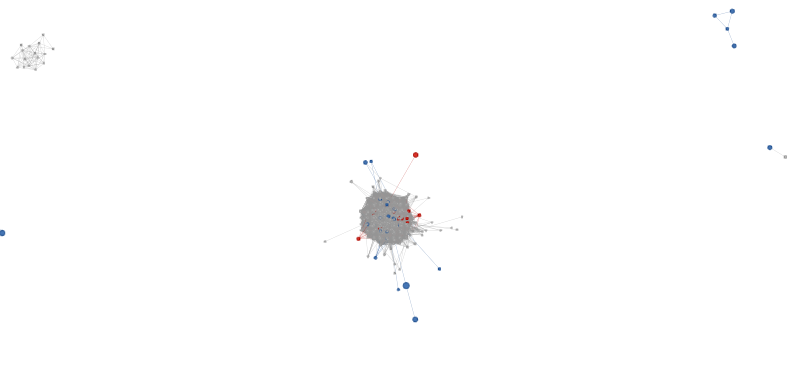

Supplement: S5 Fig — The network includes upregulated DEGs (red nodes), downregulated DEGs (blue nodes), and non-DEGs (gray nodes). The network was constructed using the 2000 most variable genes with an adjusted p-value (padj) < 0.05 and a Pearson correlation threshold of 0.7. Each node represents a gene, and each edge indicates the co-expression relationships between genes based on the correlation threshold. DEGs were identified using DESeq2 and defined as genes with a log2Fold Change ≥ 1 or ≤ −1 and padj < 0.05. The size of each node reflects the gene’s log2FoldChange. (TIFF) [file pone.0354033.s005.tiff]

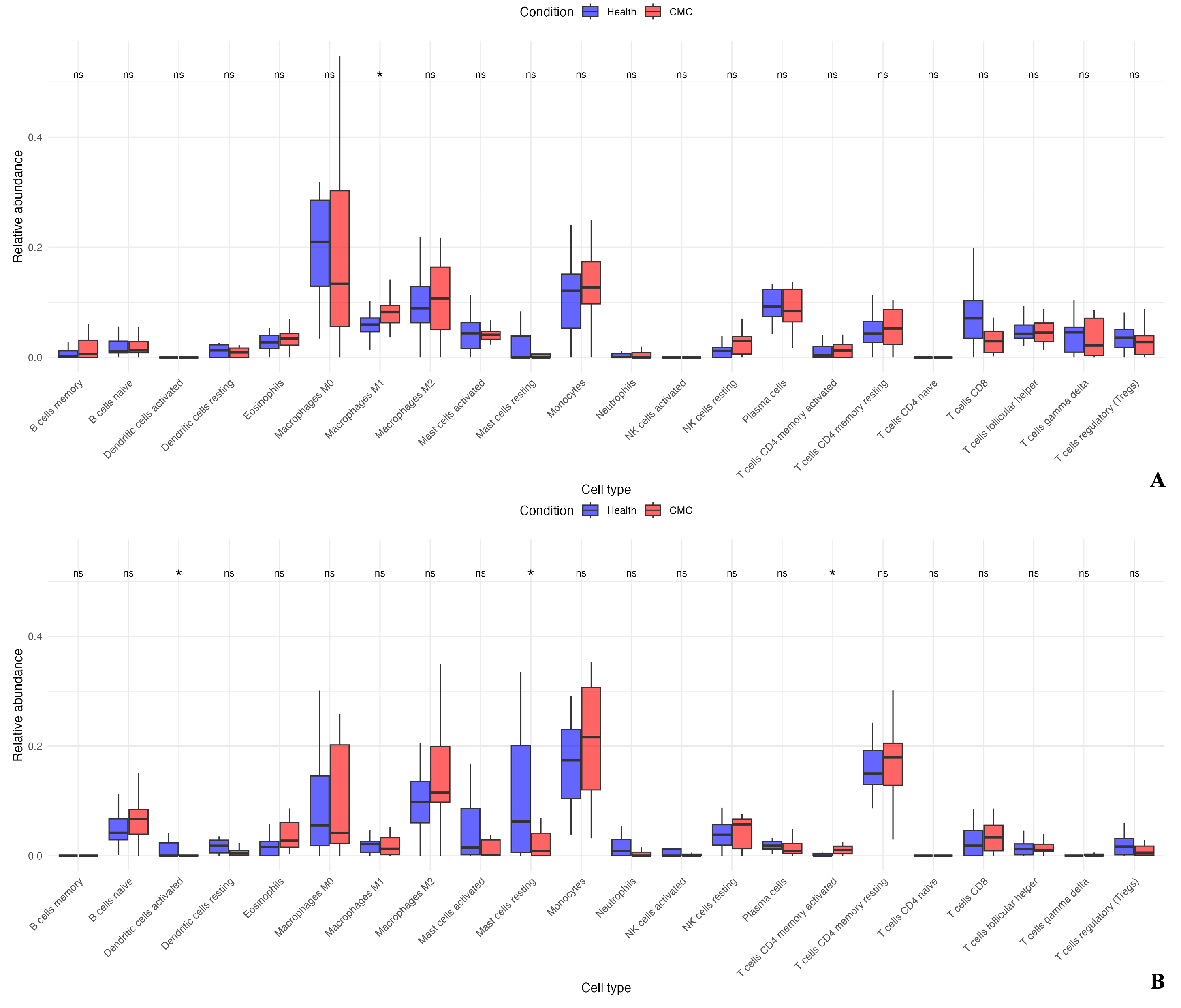

Supplement: S6 Fig — Summary of the relative proportions of 22 infiltrating immune cell types predicted from raw RNASeq counts normalized to transcripts per million (TPM) using the CIBERSORTx algorithm for the gene expression datasets GSE136197 (A) and GSE135183 (B). Statistical comparisons were performed using the Wilcoxon signed-rank test, and the p-values displayed in the figure correspond to the Wilcoxon test results (* indicates p < 0.05; ** indicates p < 0.01). p-values were additionally adjusted using the Benjamini–Hochberg false discovery rate (FDR) correction for multiple comparisons. Results that were not significant after FDR correction were interpreted as exploratory trends. (TIFF) [file pone.0354033.s006.tiff]
