# Supplementary material for: Diagnostic and prognostic values of differentially expressed genes in canine mammary carcinoma: An integrated bioinformatics analysis
Source: PLoS One. 2026 Jul 20;21(7):e0354033. doi: 10.1371/journal.pone.0354033 (PMC13384312; doi:10.1371/journal.pone.0354033)
Supplement: Attachment — Submitted filename: Response to Reviewers.pdf [file pone.0354033.s022.pdf]

Bogotá, April 2nd, 2026

**REVIEWER**
***PLOSOne***

Dear Reviewer

We are very grateful for the feedback provided by you related to the submitted manuscript entitled "***Differentially expressed genes with potential diagnostic and prognostic value in canine mammary carcinoma and their relevance for human breast cancer: an integrated bioinformatics analysis***".

Below we provide clarifications, responding to each point raised by you.

**Reviewer Recommended Revisions**

a) Immune Infiltrate Heterogeneity Claim
The manuscript states: 'Significant inter- and intratumoral heterogeneity was observed in the CMC immune infiltrate.' However, in the CPA-UN dataset no statistically significant differences were observed between tumor and healthy tissues. In GSE119810, only Tregs reached statistical significance, and in other datasets only limited immune subsets were significant. The box-and-whisker plots demonstrate variability, but do not fully support the claim of statistically significant heterogeneity across datasets. The authors should:
• Clarify whether "heterogeneity" refers to statistical significance or visual distribution variability.
• Rephrase the statement to reflect observed trends rather than broadly claiming significant heterogeneity.
• Possibly include variance metrics or formal heterogeneity testing.

The initially submitted manuscript contained an inadequate translation of the phrase "*Significant inter- and intratumoral heterogeneity was observed in the CMC immune infiltrate*." Following the recommendations, the interpretation of the results was clarified in lines 447-450 as follows: "*The observed variable behavior and trends for the 22 infiltrating immune cells inferred using CIBERSORTx between CMCs and matched adjacent healthy mammary tissues for each gene expression dataset, suggest a notable inter- and intratumoral heterogeneity in the CMC immune infiltrate*."

b) Survival Analysis Clarification
The Discussion states: 'Overexpression of 24 DEGs and underexpression of 30 DEGs were significantly linked to OS.' However, Figure 6 only displays three upregulated and three downregulated genes. To fully support the claim:
• The full list of 54 significant DEGs should be provided in a supplementary table with hazard ratios and confidence intervals.
• Figure 6 panel labels (A and B) should be clearly displayed.
• Clarify whether multiple testing correction was applied in survival analysis.

Initially for the survival analysis, the mean expression of each DEG was calculated and Kaplan-Meier and LogRank test methods were applied to compare the low and high expression survival curves. This resulted in 54 DEGs with a LogRank p-value < 0.05, suggesting that these 54 DEGs were significantly associated with OS. However, multiple testing correction was not applied.

Following the recommendation, the survival analysis was expanded to include a univariate Cox proportional hazards regression model and a multiple testing correction. In Materials and methods section, the following modifications were made:

- In lines 270-271, the phrase "*Overall survival (OS) was defined as the time in days from diagnosis to death or last follow-up*" was included.
- In lines 273-278, the paragraph "*Kaplan-Meier survival plots were generated, and a LogRank* $p < 0.05$ *was considered for identifying DEGs with nominal associations with OS. In addition, a univariate Cox proportional hazards regression model was fitted for each gene to estimate the hazard ratio (HR) and its 95% confidence interval (CI). p-values derived from the Cox models were adjusted for multiple testing using the Benjamini–Hochberg method. Genes with significant FDR-adjusted p-values were considered associated with OS*" and were included.

According to the proposed modification in the methodology, the results were modified as follows:

- Lines 463-477: "*The Cox proportional hazards regression model and Log-rank test shown 25 overexpressed DEGs with nominal associations with survival (LogRank* $p < 0.05$*) (STOML1, GADD45A, FAM20A, MIR29B-2, TM4SF5, ITGB2, DHDDS, ZCCHC17, RANBP3, TM4SF4, PSMD4, MFHAS1, S100A14, PIM1, PPBP, SFN, NELFE, KLHL38, SIDT1, VTI1B, SCPEP1, PPM1N, KRT73, SCPEP1, FYN and WASF3) whose higher expression levels were associated with an increased risk of mortality (HR > 1). Conversely, 29 underexpressed DEGs with nominal associations with survival (LogRank* $p < 0.05$*) (TRPC1, EMX2, NTSR1, CPXM2, HDGFL2, DDAH2, PRKAG2, TRIOBP, DSG1, ANKRD13A, IGFBP2, FOXP4, DOK1, QKI, MFGE8, CYP26B1, AVEN, NINJ2, ASCL2, SYN2, TMEM252, RRAGB, MPRIP, SELENOP, G6PC1, TSR3, FBLN1, CCDC152, FAM187A) were associated with a reduced risk of mortality (HR < 1), suggesting a potential protective effect. However, the 95% IC and multiple testing correction using the Benjamini–Hochberg method, shown none of the genes remained statistically significant (FDR > 0.05) (S11 Table), indicating that these findings should be interpreted as exploratory and require validation in larger cohorts. Kaplan-Meier curves of the top 3 upregulated and downregulated DEGs related to OS are shown in Fig 6.*"
- The full list of 54 DEGs with HR, IC, p-value and FDR was provided in S11 table.
- Figure 6 panel labels (A and B) was included.

In Discussion section, the following modification was made:

- Lines 594-598: "*Survival analysis of the GSE119810 dataset revealed that 25 overexpressed and 29 underexpressed DEGs were nominally linked to OS, indicating that these DEGs might serve as potential prognostic biomarkers for CMC. However, after correction for multiple testing using the Benjamini–Hochberg method, none of the genes remained statistically significant, indicating that these findings should be interpreted as exploratory and require validation in larger cohorts*."

c) Data Availability Statement

While public datasets are clearly referenced, the proprietary CPA-UN dataset does not indicate:
• GEO/SRA accession numbers
• Repository deposition plans
• Raw FASTQ availability
PLOS ONE requires full availability of underlying data. The manuscript must specify where the proprietary dataset will be deposited and provide accession numbers prior to publication.

The raw FASTQ files were submitted to the NCBI Sequence Read Archive (SRA) with the submission code SUB16061762. The BioProject was created with the code PRJNA1440953. Once the article is published, the files will be made publicly accessible.

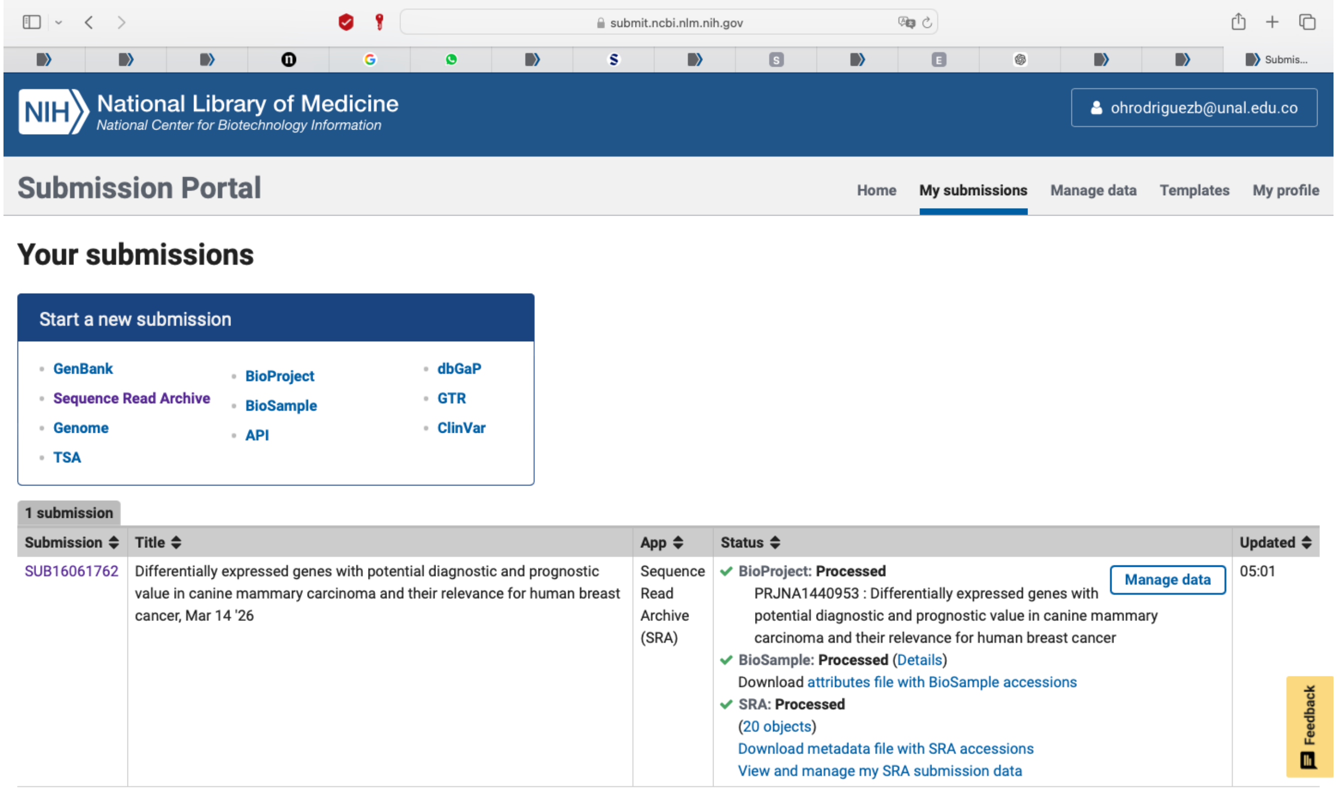


In lines 144-146, the availability of the dataset was included as follows: "*Our dataset was named "CPA-UN" and the corresponding raw RNASeq data are available on the Sequence Read Archive (SRA) section of The National Center for Biotechnology Information (NCBI) platform BioProject PRJNA557680.*"

d) Methods Clarification

• Functional enrichment (GO and KEGG) should be clearly described in the Methods section with appropriate citations.
In lines 192-200 the methodology used to perform GO and KEGG functional enrichment was included as follows: "*The list of DEGs was used as an input set in both tools and the enrichment of Gene Ontology (GO) terms in the categories Biological Process (BP), Molecular Function (MF) and Cellular Component*

*(CC) was evaluated, as well as the enrichment of metabolic pathways in Kyoto Encyclopedia of Genes and Genomes (KEGG). The analysis was performed using the enrichGO and enrichKEGG functions from the clusterProfiler and Pathview packages. p-values were calculated using hypergeometric tests and adjusted for multiple comparisons using the Benjamini–Hochberg method to control for false discovery rates (FDR). Terms with FDR-adjusted p-values (padj < 0.05) were considered significantly enriched.*"

• All bioinformatics tools (R version, packages, command-line tools) require formal citations.
The bioinformatics tools and their version used were properly referenced (references 13-25).

• Clarify normalization approach used before merging datasets for combined GSEA and co-expression network construction.
In lines 205-208 the methodology used to perform normalization in GSEA for CPA-UN dataset was included as follows: "*Additionally, a Gene Set Enrichment Analysis (GSEA) was conducted using the raw count matrix from the CPA-UN gene expression dataset, which was normalized with DESeq2 package (version 2.11.40.8) in R (Version 4.2.3) using the median of ratios method to normalize count data and produce a ranked list of DEGs generated by DESeq2 analysis as input for GSEA (Version 4.4.0)*."

In lines 214-227 the methodology used to perform the normalization of a single matrix constructed by merging the raw count tables of the 4 expression sets used in the GSEA was included as follows: "*A GSEA was also performed, combining the four gene expression data sets (CPA-UN, GSE119810, GSE136197, and GSE135183) into a single matrix. Only genes present across all datasets were retained to ensure comparability. The resulting matrix of raw counts was used as input for downstream normalization and analysis. Normalization was performed using the regularized logarithmic transformation (rlog) method and variance stabilization was performed with DESeq2 package (version 2.11.40.8) in R (Version 4.2.3). Size factors were estimated to account for differences in sequencing depth and library composition among samples, enabling accurate comparison of gene expression levels across the integrated datasets. The DESeq2 normalization framework models count data using a negative binomial distribution and applies internal scaling factors to correct for technical variability. Normalized expression values and differential expression statistics obtained from DESeq2 were subsequently used to construct a ranked list of DEGs as input for GSEA (Version 4.4.0). The molecular signatures affected by the DEGs were identified following the same methodological approach described for the GSEA of the CPA-UN gene expression data set.*"

In lines 242-248 the methodology used to perform the normalization of a single matrix constructed by merging the raw count tables of the 4 expression sets and the subsequent co-expression network analysis was included as follows: "*Additionally, a co-expression network was created by merging the four gene expression datasets (CPA-UN, GSE119810, GSE136197, and GSE135183) into a single matrix. The merged matrix of raw counts, normalization (rlog method) and variance stabilization were performed using the same methodological approach followed for the GSEA with DESeq2 package (version 2.11.40.8) in R (Version 4.2.3). From the merged matrix of normalized counts, construction and analysis of the co-expression network were performed following the same methodological approach as for the CPA-UN dataset*."

e) Minor Editorial Revisions

• Line 47: Add citation regarding increasing trend of HBC incidence.
In line 67, the citation corresponding to an updated article mentioning the increasing incidence of HBC (reference 4) was included.

• Line 48: Define TNM before first use.
In lines 68-69, TNM was defined in its first use.

• Line 83: change 'y' to 'and'.
In line 85, “y” was changed to “and”,

• Line 102: Table 1, replace 'Si' with 'Yes' for lymph node metastasis.
In line 123, “Si” was changed to “Yes” in Table 1.

• Clarify the term 'small world' in the co-expression network description for non-specialist readers.
In lines 383-384 term “small world” in the co-expression network was described as follows: “*possibly a “small-world network”, defined as a network with a high clustering coefficient and low distances between nodes*.”

• Screen entire manuscript for minor typographical inconsistencies.

We look forward to hearing from you soon.

Yours sincerely,

**Carlos Alberto Parra López, MD. PhD**
