# Supplementary material for: Diagnostic and prognostic values of differentially expressed genes in canine mammary carcinoma: An integrated bioinformatics analysis
Source: PLoS One. 2026 Jul 20;21(7):e0354033. doi: 10.1371/journal.pone.0354033 (PMC13384312; doi:10.1371/journal.pone.0354033)
Supplement: Attachment — Submitted filename: Response_to_Reviewers_auresp_2.pdf [file pone.0354033.s023.pdf]

Bogotá, June 14th, 2026

REVIEWERS
PLOSOne

Dear Reviewers

We are very grateful for the feedback you provided on the submitted manuscript titled " ***Diagnostic and prognostic values of differentially expressed genes in canine mammary carcinoma: An integrated bioinformatics analysis***".

Below, we provide clarifications, in response to each point you raised.

REVIEWER #1:

In summary, the manuscript is technically solid and suitable for publication in principle, but with the expectation that the authors make final adjustments to:

1. *Further temper conclusions especially regarding prognostic biomarkers.*
2. *Clearly and consistently frame survival results as exploratory.*
3. *Finalize and clarify data availability details.*

With these points addressed, the manuscript should be acceptable.

Dear reviewer, we sincerely thank you for carefully evaluating our manuscript and for recognizing its overall technical quality. We appreciate your constructive recommendations. We have addressed each point and included our responses in the revised manuscript. We also appreciate the reviewer's thoughtful comments, which we believe have significantly improved the manuscript's clarity, balance, and overall rigor.

1. We further moderated our conclusions throughout the manuscript, particularly regarding prognostic biomarkers. Genes identified in the analyses are now consistently described as "candidate biomarkers" or "potential biomarkers" requiring independent validation, rather than definitive markers (lines 68-73 and 824-857).

2. We agree that the survival analyses should be interpreted cautiously. Accordingly, the Results and Discussion sections were revised to clearly and consistently present the survival findings as exploratory given the relatively small sample size and the lack of statistical significance after multiple-testing correction. We also explicitly acknowledge the potential risk of false-positive findings and overfitting (lines 583-586 and lines 779-805).

3. The Data Availability Statement was revised and clarified. The raw FASTQ files have been submitted to the NCBI Sequence Read Archive (SRA) under BioProject accession PRJNA1440953 (submission code: SUB16061762). We clarified that the data will be publicly available upon publication of the manuscript.

REVIEWER #2:

The revision appears thorough and responsive. However, the title, abstract and some discussion seem to overstate their findings.

1. The title may be changed/shortened to "Diagnostic and prognostic values of differentially expressed genes in canine mammary carcinoma: an integrated bioinformatics analysis".
2. The abstract and discussion should restrain from stating too much human relevance due to the lack of substantial data.
3. The abstract's introduction should be shortened and clearly state its aims, while the results section should be expanded.

We sincerely thank these constructive comments and suggestions. We have addressed all points in the revised version.

1. We appreciate the proposed title change. The title was revised to: "*Diagnostic and prognostic values of differentially expressed genes in canine mammary carcinoma: An Integrated bioinformatics analysis*" (lines 1-3).

2. We agree that the original manuscript overemphasized its relevance to human breast cancer. According, the abstract and discussion were revised to temper statements about translational applicability and relevance to human disease. References to human breast cancer are now presented primarily in the context of comparative oncology and previously published literature rather than as direct conclusions drawn from this study (lines 28-73 and 600-821).

3. Following your recommendation, the Abstract was reorganized. The introductory section was shortened and now more clearly states the study aim. In addition, the Results section was expanded to provide a clearer summary of the principal findings, including differential expression analysis, pathway enrichment, immune infiltrate deconvolution, co-expression network, and potential candidate biomarkers (lines 28-73).

REVIEWER #3:

We sincerely thank the careful and thorough review of our manuscript and appreciate each of your constructive comments and suggestions. We have addressed all your points in the revised version.

Major comments:

1. Overstatement of translational relevance (Title and throughout the manuscript):
The manuscript suggests direct relevance to human breast cancer; however, no cross-species validation or integrative analysis with human datasets is performed. The connections to human breast cancer rely primarily on previously published literature. This overstates the translational contribution and should be moderated.

- Our intention was to highlight the potential comparative oncology value of canine mammary carcinoma rather than claim direct equivalence to human disease. In response, we revised the Title (lines 1-3), Abstract (lines 28-73), Introduction (lines 97-101), Discussion (lines 600-821), and Conclusions (lines 824-857) to moderate these statements.

2. Lack of batch effect correction in multi-dataset integration:
A central limitation of the study is the integration of multiple RNA-seq datasets without explicit batch effect correction. This issue affects several downstream analyses, including DEG identification, GSEA, and co-expression network construction. Given the heterogeneity across datasets, uncorrected technical variability may significantly bias the results and lead to misleading biological interpretations.

- Batch correction was performed using ComBat-seq (SVA package), with dataset origin as the batch variable, although this was not explicitly stated in the original manuscript. This information has been added

to the Materials and Methods section, and the Discussion was expanded to acknowledge the possibility of residual technical variability despite batch correction (lines 257-260 and 620-623).

3. DEG filtering strategy and exclusion of non-coding genes:

The exclusion of genes with very high counts is not standard practice and lacks justification, potentially removing biologically relevant signals. Additionally, the removal of non-coding genes limits the scope of the analysis by excluding important regulatory elements such as polyadenylated lncRNAs.

- The filtering strategy was revised and clarified in the Materials and methods section. This filtering strategy increased the number of DEGs across all datasets. Nevertheless, the functional enrichment analysis yielded results consistent with those reported using the previous filtering approach. In contrast, the inclusion of LOC genes modified the Results and Discussion of both the co-expression network and survival analyses. S4 Table has also been added, listing the predicted/uncharacterized genes annotated with LOC identifiers and their corresponding gene biotypes, as available in the GTF file (lines 211–213 and 352-356).

4. Immune infiltrate deconvolution limitations:

The use of the LM22 signature matrix, derived from human data, may introduce bias when applied to canine samples. Furthermore, multiple statistical comparisons were performed without clear correction for multiple testing, increasing the risk of false-positive findings. Many reported observations are based on non-significant trends, which should be interpreted cautiously.

- We acknowledge that the LM22 signature matrix was derived from human leukocyte expression profiles and may not fully represent the immune landscape of canine carcinomas. However, due to the limited availability of validated canine-specific reference matrices, LM22 has previously been used as an exploratory approach in canine cancers ((doi: 10.1038/s42003-023-05208-z, doi: 10.3389/fvets.2023.1192525, doi: 10.1016/j.ccell.2020.01.004). Therefore, we applied this method as an exploratory analysis and explicitly instated in that the CIBERSORTx results should be interpreted cautiously as estimates rather than absolute quantifications. This limitation was also addressed in the Discussion section (lines 302-308, 313-318, and 770–777).

- The immune deconvolution analysis in the Methods section was revised to improve statistical rigor. The paired Student's t test was replaced with the Wilcoxon rank-sum test, and p-values were adjusted using the Benjamini–Hochberg correction. The Results and Discussion sections were revised to distinguish statistically significant findings from non-significant trends, which are now described more cautiously as exploratory observations ((lines 313–321, 530-557, and 752-762).

5. Co-expression network analysis concerns:

The construction of co-expression networks without correcting for batch effects is particularly problematic, as correlation-based methods are highly sensitive to technical variation. Restricting the network to DEGs may also bias hub gene identification. Additionally, the classification of networks as "small-world" is not sufficiently supported without comparison to appropriate null models. Inconsistencies in reporting (e.g., "top 10" hub genes including more than 10 genes) further reduce clarity.

- We appreciate these important methodological observations. As noted above, batch correction was performed using ComBat-seq. Initially, the network was intentionally constructed using DEGs to focus on transcriptional alterations associated with CMC, while recognizing that this approach may influence hub-gene identification and does not capture the full transcriptomic network. We have now adopted an unsupervised,

variance-based approach, and the number of genes included in the network was determined using the elbow method applied to the variance distribution. Differential expression results were used only for node annotation and were not involved in network construction or hub gene identification. The term “small-world” was removed from the Results section. The co-expression network analysis was revised to report the network construction strategy, key topological metrics, module-specific hub genes, and co-expression modules. The manuscript was revised to correct reporting inconsistencies, including the inaccurate reference to “top 10” hub genes (lines 275-298 and 465-524).

6. ***GSEA and functional enrichment interpretation:***

While enrichment analyses identify relevant biological processes, the integration of multiple datasets without correction may bias GSEA results. The interpretation would benefit from a more integrated and critical discussion rather than descriptive reporting.

- We thank you for this constructive suggestion. As noted earlier, batch correction was performed before downstream analyses, including GSEA. The discussion was revised to offer a more integrative and critical interpretation of the GSEA results, emphasizing recurrent biological themes across datasets rather than individual enriched pathways. Interpretations were also moderated, and pathway enrichment is now described as transcriptomic patterns rather than direct evidence of functional activation (lines 704-723).

7. ***Survival analysis limitations:***

The survival analysis is limited by a relatively small sample size (n = 47), which likely reduces statistical power. Although several genes show nominal significance, none remain significant after multiple testing correction, suggesting a high likelihood of false-positive findings. These results should be clearly framed as exploratory, and the potential for overfitting should be addressed.

- In the revised manuscript, the survival analysis is now explicitly described as exploratory. The revised gene-filtering strategy altered the number of DEGs included in the analyses, necessitating in corresponding updates to the Results, which have been revised throughout the manuscript. We clarify that although several genes showed nominal associations with survival outcomes, none remained statistically significant after multiple-testing correction. Accordingly, in the Discussion, these genes are now presented as preliminary prognostic candidates requiring validation in larger independent cohorts ((lines 332-339, 565-582, and 779-805).

8. ***Overinterpretation of candidate biomarkers:***

The identification of seven shared DEGs is interesting; however, given the limited overlap across heterogeneous datasets and absence of experimental validation, their roles as “diagnostic” or “crucial” biomarkers are overstated. These should be presented as candidate genes requiring further validation.

- The manuscript has been revised to refer to these genes as “candidate genes” or “candidate biomarkers” rather than validated diagnostic markers. We also emphasize the limited overlap among datasets and the lack of experimental validation, and we have moderated the conclusions accordingly (lines 600-857).

9. ***Integration of results in the discussion:***

Although the discussion attempts to integrate multiple analytical layers, it does not sufficiently account for dataset variability and methodological limitations. This leads to an overextended interpretation of findings, particularly regarding immune infiltration patterns and pathway activation.

- The Discussion was revised to more explicitly acknowledge dataset variability and methodological limitations throughout the interpretation of the results. We moderated the discussion of immune infiltration patterns, pathway enrichment, and candidate genes, emphasizing their exploratory nature and the need for further validation (lines 600-821).

Minor comments:

- Introduction - clarity of novelty:
The introduction would benefit from a clearer definition of the specific knowledge gap addressed by this study and how it advances beyond existing transcriptomic analyses.

- We thank you for this insightful comment. The Introduction was revised to more clearly define the specific knowledge gap this study addresses and to better highlight its novelty. A paragraph addressing this suggestion was added (lines 103-108). Additionally, the introduction was enhanced with further suggestions from reviewer #4.

- Methods - sample handling details:
Additional details regarding tissue fragment size and RNAlater volume relative to tissue mass would improve reproducibility.

- To improve methodological clarity and reproducibility, additional details on tissue handling and preservation were added to into the Materials and methods section (lines 135-142).

- RNA-seq pipeline – strandedness:
The absence of explicit strandedness parameters during quantification may affect gene-level accuracy, particularly for overlapping transcripts.

- Library preparation was performed using the TruSeq Stranded Total RNA Sample Preparation Kit, which generates strand-specific libraries. Accordingly, the appropriate strandedness parameters used during quantification have now been explicitly include in the Methods section to improve transparency and reproducibility (lines 200-202).

- DEG variability across datasets:
The large variation in the number of DEGs identified across datasets should be discussed more explicitly, as it may reflect technical or biological heterogeneity.

We appreciate this important observation. The revised Discussion now acknowledges that the variability in the number of DEGs across datasets likely reflects both biological variability and technical differences among studies, including sample collection, tissue handling, RNA quality, sequencing protocols, and bioinformatic workflows (lines 600-821).

- Functional enrichment presentation:
Results are largely presented as lists of enriched terms; a more synthesized biological interpretation would improve readability and impact.

- In the Discussion section, a paragraph was added to revise the functional enrichment results providing a more integrated biological interpretation rather than primarily listing significantly enriched terms and pathways (lines 674-702).

- Downregulated gene interpretation:

The enrichment of muscle-related processes among downregulated genes is biologically meaningful but insufficiently explored.

- In the Discussion section, a paragraph was added to expand in which the interpretation of the downregulated gene enrichment results (lines 687-698).

- Redundancy in GSEA results:
Some pathways are reported multiple times and should be consolidated for clarity.

- In the results section, redundant and highly overlapping GSEA pathways between the CPA-UN dataset and the combined dataset were consolidated. Pathways specific to each dataset were also noted. Additionally, in the Discussion section, a paragraph was added to present broader functional categories and integrated biological themes to improve clarity and readability (lines 441-455 and 704-723).

- Immune infiltrate interpretation:
Non-significant findings should be clearly distinguished from statistically supported results to avoid overinterpretation.

- We thank you for this important observation and agree that non-significant findings should be interpreted cautiously. The Results and Discussion sections on the immune infiltrate were revised to clearly distinguish statistically supported results from non-significant trends. Findings that did not remain significant after FDR correction are now described as exploratory trends rather than definitive alterations in immune infiltration (lines 530-557 and 752-762).

- Reporting inconsistencies:
There are inconsistencies in reporting hub genes (e.g., "top 10" lists exceeding 10 genes) and minor issues such as duplicated gene names that should be corrected.

We thank you for identifying these inconsistencies. The manuscript was revised to correct reporting inconsistencies, including the inaccurate reference to "top 10" hub genes (lines 480-524).

- Conclusion – tone adjustment:
The conclusions should be moderated to reflect the exploratory nature of the findings and the lack of validation, particularly regarding biomarker claims and relevance to human breast cancer.

We thank you for this important comment. The Conclusions and Future Perspectives section was revised to better reflect the exploratory nature of the findings and the absence of experimental validation. Candidate genes are now described more cautiously as potential biomarkers that require further validation in independent cohorts and through functional studies. In addition, statements about relevance to human breast cancer were moderated (lines 824- 857).

REVIEWER #4:

Dear reviewer, we sincerely thank you for your careful and thorough review of our manuscript and appreciate each of the constructive comments and suggestions you have made. We have addressed all points in the revised version.

- Abstract
Abstract requires major revision. There is no clear description of RNAseq data CPA-UN, which is confusing and lacks transparency. A vague and brief reference to "a proprietary dataset" is not sufficient.

The terminology over/under expressed is technically inaccurate and should be changed to reflect the comparative outcome that is being measured, i.e. transcript levels were higher or lower in one group compared to another. I agree with the previous reviewer that using the term heterogeneity is confusing when no distribution analyses between studies were completed or differences between groups have been found. Variation within and between would be expected. Additionally, the variation is not likely all biological. There are likely components due to sample collection, handling, and preservation, etc. The conclusion statement should be adjusted to reflect these results require further investigation before any definitive interpretations can be made. Furthermore, the limitations of secondary data analysis and likely contributions tissue collection and handling issues should be added.

- The description of the "CPA-UN" RNA-seq dataset was clarified, and it is now explicitly stated that it is an independently generated RNA-seq dataset produced in the present study. The terms "overexpressed" and "underexpressed" were replaced with more accurate comparative descriptions. The presentation of the summarized results from the immune infiltrate deconvolution was revised to avoid confusion associated with the term "heterogeneity". The concluding statements were moderated to emphasize the exploratory nature of the findings and the need for additional validation, and the limitations associated with secondary transcriptomic data integration. Potential pre-analytical and technical sources of variation, were also incorporated (lines 28-73).

- Introduction

Brief and concise. Some of the ideas/topics would fit better in the discussion section. What is meant by "close relationship" between dogs and humans should be more clearly defined.

- The Introduction was revised to improve focus and clarity by removing or moderating broader interpretive statements, clarifying the phrase about the "close relationship" between dogs and humans, and further emphasizing the study´s exploratory nature and the need for additional validation in larger independent cohorts and future functional studies (lines 97-101).

- Methods

Methods are inclusive of info required for this work to be reproduced. This section could be organized better. Furthermore, additional consideration toward data cleaning may impact the outcome of your work.

Data handling/cleaning is not clear. There was quality control of RNA; however, it was not stated if data were cleaned to remove tissue specific genes that do not belong in mammary glands i.e. muscle tissue, skin tissue, which are easy to contaminate MG samples. This is highly relevant to the outcome of this study, as the results suggest problems with data quality.

- In the present study, tissue sampling was performed exclusively from the tumor region by the veterinary surgeon during mastectomy, and careful dissection was conducted to avoid including surrounding skin or muscle tissue. This clarification has now been incorporated into the Methods section. Nevertheless, we acknowledge that despite careful sampling procedures, some degree of tissue heterogeneity is difficult to completely exclude in bulk RNA-seq studies and should be considered when interpreting the results (lines 135-138).

A more compelling rationale is needed to justify including the data set GSE135183, as this RNAseq project used RNA extracted from stroma excised by LCMD from FFPE tissues. All other RNAseq projects in this study used frozen carcinoma tissues: Therefore, the data in GSE135183 are from a tissue subtype, not whole tissue. Furthermore, the tissues were preserved differently. This must be considered and put into context.

- The GSE135183 dataset was included because it was generated using comparable Illumina NGS technology and provided paired adjacent mammary tissue samples for each carcinoma case, which was consistent with the study design used for the other datasets. We acknowledge that using LCMD stromal FFPE tissue may introduce biological and technical variability, and this limitation is now discussed more clearly in the revised manuscript. In addition, the Methods section was updated to explicitly describe these characteristics of the GSE135183 dataset (Lines 185-187 and 606-637).

More compelling rationale for integrating versus analyzing data separately should be provided either in the methods or results section.

- In the Methods section of the revised manuscript, we clarified that the datasets were initially analyzed separately to preserve the biological and technical characteristics of each independent cohort, particularly the CPA-UN dataset generated in the present study. Subsequently, integrative analyses were conducted to identify recurrent DEGs, pathways, and co-expression patterns consistently observed across independent CMC datasets, thereby increasing the robustness of the findings despite inter-dataset variability (lines 208-213, 223-229, and 275-280).

I suggest a separate data handling and statistical analysis section.

- We carefully considered creating a separate Data Handling and Statistical Analysis section; however, we decided to keep these descriptions within the corresponding methodological subsections to preserve the manuscript´s workflow and readability. To improve clarity, further clarify and expanded the relevant data processing, normalization, batch-correction, and statistical procedures in the appropriate sections of the Methods.

- Results

Pathway analysis results represented in figure 2 are misreported in the results section. Results in Figure 2a strongly suggest that at least some of the carcinoma tissue samples were contaminated with muscle. Nine out of ten biological processes listed are muscle associated. The text of the results section reports only nine "top 10 enriched and significant biological process" and none of the ten categories listed in figure 2a when referring to "significant biological process" on lines 314-319. p-value rankings and color associations are inconsistent across Figure 2 panels. Figure S1 panels in the figure and those indicated in the corresponding legend disagree. Figure 2 contains results of downregulated DEGs. In other words, Figures 2 and S1 contain the same results!

- We sincerely thank you for identifying these inconsistencies and apologize for the errors in the original manuscript. It is correct that the figure initially presented as Fig 2 mistakenly displayed enrichment results for downregulated DEGs, resulting in duplication with S1 Fig. This also created inconsistencies between the Results section and the figure panels. These issues have now been corrected throughout the revised manuscript, including the replacement of the correct Fig. 2 and the revision of the corresponding Results section (lines 394-421).

Figure 1, fyi, excluding GSE135183 adds three more increased expression genes to the "in common with all data sets" category. One of these, MIA, is an interesting cancer gene.

- We thank you for this insightful observation. We agree that excluding GSE135183 identifies additional shared genes, including MIA. Accordingly, we also explored Venn diagrams using three alternative dataset combinations, which revealed several additional shared DEGs. However, we applied a conservative criterion in the main analysis by restricting shared genes to those consistently identified across all four datasets to prioritize robustness and reproducibility. Additional discussion of these alternative dataset combinations was incorporated into the Discussion section (lines 371-383 and 666-672).

Line 358, “E2F targets” is repeated.

- We thank you for identifying this duplication. The repeated mention of “E2F targets” has now been corrected in the revised manuscript.

Line 361, “glycolysis” is not evident in figure 3.

- We appreciate the identification of this inconsistency. This discrepancy has now been corrected in the revised manuscript by ensuring consistency between the text and the pathways displayed in the figure (lines 441-460).

Figures 3, 4, Compelling rationale for analyzing CPA-UN separately and then integrated with the three GSE datasets was not provided.

- The revised manuscript now clarifies that the CPA-UN dataset was initially analyzed separately to characterize the independently generated RNASeq cohort produced in the present study and to preserve transparency regarding the primary data. Subsequent integrative analyses with the GEO datasets were performed to identify recurrent transcriptional signatures consistently observed across independent cohorts. Therefore, additional clarification was incorporated into the Methods sections (lines 225-231 and 277-282).

Figure 5 A is null data and therefore not necessary. In the corresponding text: lines 427-430 it is reported that there were no significant differences. This should be the end of the results story here; therefore, it is inappropriate to report lines 430 – 433 that there was an increase in M1, NKs, and plasma, and a decrease M2, CD4 and CD8 Ts. This must be removed. It would be appropriate to present these trends in the discussion section as a comparison with the other RNAseq data sets used.

- We thank you for these thoughtful comments. Accordingly, statements describing increases or decreases in immune cell populations without statistical significance were removed from the Results section and are now discussed more cautiously as exploratory trends. Regarding Figure 5A, we decided to retain this panel because it corresponds to the independently generated CPA-UN dataset produced in the present study. However, both the figure and the text were revised to clearly emphasize the exploratory nature of these findings and the absence of statistically significant differences (lines 530-557 and 752-762).

Figure 5B, the asterisks indicating significant differences for Tregs was difficult to see and should be moved or made larger. Same for Figure S8.

- We thank you for identifying this visualization issue. In the revised manuscript, the asterisks indicating statistically significant differences for specific cells in Figure 5B and Supplementary Figure S6 were repositioned and enlarged to improve readability and clarity.

Line 455, a Student’s t test here is not conservative and does not correct for multiple comparisons. This should be an ANOVA followed by an appropriate post hoc test to compare groups if the F test is significant. Alternatively, it may be more appropriate to use a nonparametric statistical method here. Same for Figure S8.

- We thank you for this important methodological observation. The original paired Student’s t test was replaced with the nonparametric Wilcoxon signed-rank test, and p-values were adjusted using the Benjamini–Hochberg FDR correction for multiple comparisons. These changes were applied to both Figure 5 and Supplementary Figure S6, and the revised statistical methodology has been incorporated into the Methods section (lines 316-321, 553-557, and 923-927).

Compelling rationale for analyzing all datasets separately for deconvolution analysis needs to be provided.

- The rationale for analyzing each dataset independently was clarified in the manuscript Immune deconvolution was performed separately for each dataset to account for differences in sample composition, tissue preservation, sequencing protocols, and cohort characteristics, thereby minimizing technical heterogeneity and avoiding potential biases associated with dataset integration. This approach also enabled comparison of immune infiltration patterns across independent datasets (lines 302-308).

Lines 473 – 477, if none of these gene differences were statistically significant after the appropriate statistical testing procedure, they should not be presented as significantly different in Figure 6.

- We thank you for this important observation. Accordingly, Fig 6 and the corresponding Results section were revised to clearly indicate that these findings represent exploratory survival associations. We now explicitly state that although several genes showed nominal significance in the LogRank analysis, none remained statistically significant after FDR correction. Therefore, these genes are presented as preliminary prognostic candidates requiring validation in larger independent cohorts (lines 586-588 and 591-597).

Lines 476, 480, 481, a more detailed description of how the “top” genes were selected for figure 6 is required.

- The revised manuscript now clarifies that the genes shown in Fig. 6 were selected based on the lowest nominal LogRank p-values and the highest hazard ratios (HRs) among the upregulated and downregulated DEGs identified in the exploratory survival analysis (lines 586-588 and 591-597).

- Discussion section:

The discussion lacks depth and impact.

Much of the first paragraph of the discussion section is introductory material and could be removed or moved to the introduction. Repetition of the methods and results makes up too much of the discussion section. The genes Lines 691-692, I suggest changing the wording or presentation here. There is no real clinical value in gene expression signatures as potential diagnostic biomarkers for tumor bearing animals. More discussion regarding the nonoverlap between RNAseq database used is warranted as this work highlights the need for robust and rigorous studies to gain reliable data and information regarding CMC. Discussion of the biopsy and sample handling differences in the context of this and future studies is required. The value this study is that it highlights the problems of working with mammary carcinoma tissue, the limitations of small sample sizes, and an alarming level of nonoverlap between studies.

We thank you for these insightful comments. The Discussion was revised to reduce introductory content and repetition of methods and results, while strengthening the interpretation of the findings and their limitations. Statements suggesting direct diagnostic or clinical utility were toned down, and the identified genes are now presented as candidate molecular markers requiring further validation. We also expanded the discussion of inter-dataset heterogeneity, limited DEG overlap, and the potential impact of differences in tissue sampling, sample handling, preservation methods, and sequencing protocols. Finally, the limitations section was strengthened to emphasize the challenges associated with CMC transcriptomic studies, including small sample sizes, dataset variability, and the need for standardized approaches and independent validation (lines 600-821).

We look forward to hearing from you.

Yours sincerely,

Carlos Alberto Parra López, MD. PhD
