# Supplementary material for: Diagnostic and prognostic values of differentially expressed genes in canine mammary carcinoma: An integrated bioinformatics analysis
Source: PLoS One. 2026 Jul 20;21(7):e0354033. doi: 10.1371/journal.pone.0354033 (PMC13384312; doi:10.1371/journal.pone.0354033)
Supplement: Peer review history

# Peer review: Decision Letter 0

**DOI:** 10.1371/journal.pone.0354033.r001

**Authors:** Xin Sun

6 Mar 2026

PONE-D-25-61057

DIFFERENTIALLY EXPRESSED GENES WITH POTENTIAL DIAGNOSTIC AND PROGNOSTIC VALUE IN CANINE MAMMARY CARCINOMA AND THEIR RELEVANCE FOR HUMAN BREAST CANCER: AN INTEGRATED BIOINFORMATICS ANALYSIS.

PLOS One

Dear Dr. Parra-López,

Thank you for submitting your manuscript to PLOS ONE. After careful consideration, we feel that it has merit but does not fully meet PLOS ONE’s publication criteria as it currently stands. Therefore, we invite you to submit a revised version of the manuscript that addresses the points raised during the review process.

Please note that we have only been able to secure a single reviewer to assess your manuscript. We are issuing a decision on your manuscript at this point to prevent further delays in the evaluation of your manuscript. Please be aware that the editor who handles your revised manuscript might find it necessary to invite additional reviewers to assess this work once the revised manuscript is submitted. However, we will aim to proceed on the basis of this single review if possible.

Please submit your revised manuscript by Apr 20 2026 11:59PM. If you will need more time than this to complete your revisions, please reply to this message or contact the journal office at plosone@plos.org. When you're ready to submit your revision, log on to https://www.editorialmanager.com/pone/ and select the 'Submissions Needing Revision' folder to locate your manuscript file.

Please include the following items when submitting your revised manuscript.

- A letter that responds to each point raised by the academic editor and reviewer(s). You should upload this letter as a separate file labeled 'Response to Reviewers'.
- A marked-up copy of your manuscript that highlights changes made to the original version. You should upload this as a separate file labeled 'Revised Manuscript with Track Changes'.
- An unmarked version of your revised paper without tracked changes. You should upload this as a separate file labeled 'Manuscript'.

If you would like to make changes to your financial disclosure, please include your updated statement in your cover letter. Guidelines for resubmitting your figure files are available below the reviewer comments at the end of this letter.

If applicable, we recommend that you deposit your laboratory protocols in protocols.io to enhance the reproducibility of your results. Protocols.io assigns your protocol its own identifier (DOI) so that it can be cited independently in the future. For instructions see: https://journals.plos.org/plosone/s/submission-guidelines#loc-laboratory-protocols. Additionally, PLOS ONE offers an option for publishing peer-reviewed Lab Protocol articles, which describe protocols hosted on protocols.io. Read more information on sharing protocols at https://plos.org/protocols?utm_medium=editorial-email&utm_source=authorletters&utm_campaign=protocols.

We look forward to receiving your revised manuscript.

Kind regards,

Xin Sun, PhD

Staff Editor

PLOS One

Journal Requirements:

When submitting your revision, we need you to address these additional requirements.

1. Please ensure that your manuscript meets PLOS ONE's style requirements, including those for file naming. The PLOS ONE style templates can be found at

https://journals.plos.org/plosone/s/file?id=wjVg/PLOSOne_formatting_sample_main_body.pdf and

https://journals.plos.org/plosone/s/file?id=ba62/PLOSOne_formatting_sample_title_authors_affiliations.pdf

2. Thank you for stating the following in the Acknowledgments Section of your manuscript:

“This work was supported by grant funding from the Ministry of Science, Technology, and Innovation of Colombia (MinCiencias), Program 92191, Project 92268-Contract No. 800-2023.”

We note that you have provided funding information that is not currently declared in your Funding Statement. However, funding information should not appear in the Acknowledgments section or other areas of your manuscript. We will only publish funding information present in the Funding Statement section of the online submission form.

Please remove any funding-related text from the manuscript and let us know how you would like to update your Funding Statement. Currently, your Funding Statement reads as follows:

“This work was supported by grant funding from the Ministry of Science, Technology, and Innovation of Colombia (MinCiencias), Program 92191, Project 92268-Contract No. 800-2023.”

Please include your amended statements within your cover letter; we will change the online submission form on your behalf.

3. Thank you for stating the following financial disclosure:

“This work was supported by grant funding from the Ministry of Science, Technology, and Innovation of Colombia (MinCiencias), Program 92191, Project 92268-Contract No. 800-2023.”

Please state what role the funders took in the study.  If the funders had no role, please state: "The funders had no role in study design, data collection and analysis, decision to publish, or preparation of the manuscript."

If this statement is not correct you must amend it as needed.

Please include this amended Role of Funder statement in your cover letter; we will change the online submission form on your behalf.

4. Please remove your figures from within your manuscript file, leaving only the individual TIFF/EPS image files, uploaded separately. These will be automatically included in the reviewers’ PDF.

5. Please include captions for your Supporting Information files at the end of your manuscript, and update any in-text citations to match accordingly. Please see our Supporting Information guidelines for more information: http://journals.plos.org/plosone/s/supporting-information.

6. If the reviewer comments include a recommendation to cite specific previously published works, please review and evaluate these publications to determine whether they are relevant and should be cited. There is no requirement to cite these works unless the editor has indicated otherwise.

[Note: HTML markup is below. Please do not edit.]

Reviewers' comments:

Reviewer's Responses to Questions

Comments to the Author

1. Is the manuscript technically sound, and do the data support the conclusions?

The manuscript must describe a technically sound piece of scientific research with data that supports the conclusions. Experiments must have been conducted rigorously, with appropriate controls, replication, and sample sizes. The conclusions must be drawn appropriately based on the data presented.

Reviewer #1: Partly

**********

2. Has the statistical analysis been performed appropriately and rigorously?

Reviewer #1: Yes

**********

3. Have the authors made all data underlying the findings in their manuscript fully available?

The PLOS Data policy requires authors to make all data underlying the findings described in their manuscript fully available without restriction, with rare exception (please refer to the Data Availability Statement in the manuscript PDF file). The data should be provided as part of the manuscript or its supporting information, or deposited to a public repository. For example, in addition to summary statistics, the data points behind means, medians and variance measures should be available. If there are restrictions on publicly sharing data—e.g. participant privacy or use of data from a third party—those must be specified.

Reviewer #1: No

**********

4. Is the manuscript presented in an intelligible fashion and written in standard English?

PLOS ONE does not copyedit accepted manuscripts, so the language in submitted articles must be clear, correct, and unambiguous. Any typographical or grammatical errors should be corrected at revision, so please note any specific errors here.

Reviewer #1: Yes

**********

5. Review Comments to the Author

Please use the space provided to explain your answers to the questions above. You may also include additional comments for the author, including concerns about dual publication, research ethics, or publication ethics. (Please upload your review as an attachment if it exceeds 20,000 characters)

Reviewer #1: This manuscript presents original research investigating differentially expressed genes (DEGs) in canine mammary carcinoma (CMC) using an integrated RNASeq bioinformatics approach that combines a proprietary dataset with three GEO datasets. The study is technically well-structured and addresses a relevant translational oncology question within a comparative One Health framework.

Strengths

• The study presents original integrated transcriptomic analysis in CMC.

• Results have not been previously published elsewhere.

• Bioinformatics workflows are appropriate and use standard, validated tools (DESeq2, HISAT2, HTSeq, ClusterProfiler, GSEA, CIBERSORTx).

• Conclusions are generally supported by the data presented.

• The manuscript is clearly written and well organized.

• Ethical approval and research integrity standards are appropriately documented.

• The study does not present dual-use concerns.

Recommended Revisions

a) Immune Infiltrate Heterogeneity Claim

The manuscript states: ‘Significant inter- and intratumoral heterogeneity was observed in the CMC immune infiltrate.’

However, in the CPA-UN dataset no statistically significant differences were observed between tumor and healthy tissues. In GSE119810, only Tregs reached statistical significance, and in other datasets only limited immune subsets were significant.

The box-and-whisker plots demonstrate variability, but do not fully support the claim of statistically significant heterogeneity across datasets. The authors should:

• Clarify whether “heterogeneity” refers to statistical significance or visual distribution variability.

• Rephrase the statement to reflect observed trends rather than broadly claiming significant heterogeneity.

• Possibly include variance metrics or formal heterogeneity testing.

b) Survival Analysis Clarification

The Discussion states: ‘Overexpression of 24 DEGs and underexpression of 30 DEGs were significantly linked to OS.’

However, Figure 6 only displays three upregulated and three downregulated genes.

To fully support the claim:

• The full list of 54 significant DEGs should be provided in a supplementary table with hazard ratios and confidence intervals.

• Figure 6 panel labels (A and B) should be clearly displayed.

• Clarify whether multiple testing correction was applied in survival analysis.

c) Data Availability Statement

While public datasets are clearly referenced, the proprietary CPA-UN dataset does not indicate:

• GEO/SRA accession numbers

• Repository deposition plans

• Raw FASTQ availability

PLOS ONE requires full availability of underlying data. The manuscript must specify where the proprietary dataset will be deposited and provide accession numbers prior to publication.

d) Methods Clarification

Functional enrichment (GO and KEGG) should be clearly described in the Methods section with appropriate citations.

All bioinformatics tools (R version, packages, command-line tools) require formal citations.

Clarify normalization approach used before merging datasets for combined GSEA and co-expression network construction.

e) Minor Editorial Revisions

• Line 83: change 'y' to 'and'.

• Line 102: Table 1, replace 'Si' with 'Yes' for lymph node metastasis.

• Line 48: Define TNM before first use.

• Line 47: Add citation regarding increasing trend of HBC incidence.

• Clarify the term 'small world' in the co-expression network description for non-specialist readers.

• Screen entire manuscript for minor typographical inconsistencies.

In conclusion, the manuscript presents a technically sound and relevant comparative oncology study. The conclusions are generally supported by the data, with minor overinterpretation in the immune infiltration section and insufficient display of survival analysis data.

These concerns are addressable through minor revision, and the study has strong potential for publication following clarification.

**********

6. PLOS authors have the option to publish the peer review history of their article (what does this mean?). If published, this will include your full peer review and any attached files.

If you choose “no”, your identity will remain anonymous but your review may still be made public.

Do you want your identity to be public for this peer review? For information about this choice, including consent withdrawal, please see our Privacy Policy.

Reviewer #1: Yes: Farruk Kabir

**********

[NOTE: If reviewer comments were submitted as an attachment file, they will be attached to this email and accessible via the submission site. Please log into your account, locate the manuscript record, and check for the action link "View Attachments". If this link does not appear, there are no attachment files.]

To ensure your figures meet our technical requirements, please review our figure guidelines: https://journals.plos.org/plosone/s/figures

You may also use PLOS’s free figure tool, NAAS, to help you prepare publication quality figures: https://journals.plos.org/plosone/s/figures#loc-tools-for-figure-preparation.

NAAS will assess whether your figures meet our technical requirements by comparing each figure against our figure specifications.

# Author comment: Author response to Decision Letter 1

**DOI:** 10.1371/journal.pone.0354033.r002

2 Apr 2026

Dear Editor and Reviewer

Below we provide clarifications, responding to each point raised by the academic editor and the reviewer.

Journal Requirements:

1. Please ensure that your manuscript meets PLOS ONE's style requirements, including those for file naming.

The manuscript was adjusted according to the style requirements regarding Article Title, Author Signature, Affiliations, Corresponding Authorship, Contribution, Level Headings, Figure Citations, Figure Captions, Tables, and Table Citations.

2. Thank you for stating the following in the Acknowledgments Section of your manuscript: “This work was supported by grant funding from the Ministry of Science, Technology, and Innovation of Colombia (MinCiencias), Program 92191, Project 92268-Contract No. 800-2023.”. Please remove any funding-related text from the manuscript and let us know how you would like to update your Funding Statement.

Funding information was removed from the manuscript. Please, modify the Funding Statement as follows: “This work was supported by grant funding from the Ministry of Science, Technology, and Innovation of Colombia (MinCiencias), Program 92191, Project 92268-Contract No. 800-2023. The funders had no role in study design, data collection and analysis, decision to publish, or preparation of the manuscript.”

3. Thank you for stating the following financial disclosure: “This work was supported by grant funding from the Ministry of Science, Technology, and Innovation of Colombia (MinCiencias), Program 92191, Project 92268-Contract No. 800-2023.” Please state what role the funders took in the study. If the funders had no role, please state: "The funders had no role in study design, data collection and analysis, decision to publish, or preparation of the manuscript."

Please, modify the Funding Statement as follows: “This work was supported by grant funding from the Ministry of Science, Technology, and Innovation of Colombia (MinCiencias), Program 92191, Project 92268-Contract No. 800-2023. The funders had no role in study design, data collection and analysis, decision to publish, or preparation of the manuscript.”.

4. Please remove your figures from within your manuscript file, leaving only the individual TIFF/EPS image files, uploaded separately. These will be automatically included in the reviewers’ PDF.

The figures were removed from the manuscript and from the Supplementary figures.docx archive.

5. Please include captions for your Supporting Information files at the end of your manuscript, and update any in-text citations to match accordingly.

The Supporting Information section was included at the end of the manuscript, including the respective captions, and the respective citations in the text were updated accordingly.

6. If the reviewer comments include a recommendation to cite specific previously published works, please review and evaluate these publications to determine whether they are relevant and should be cited. There is no requirement to cite these works unless the editor has indicated otherwise.

The reviewer did not include a recommendation to cite specific previously published works.

Reviewer Recommended Revisions

a) Immune Infiltrate Heterogeneity Claim

The manuscript states: ‘Significant inter- and intratumoral heterogeneity was observed in the CMC immune infiltrate.’ However, in the CPA-UN dataset no statistically significant differences were observed between tumor and healthy tissues. In GSE119810, only Tregs reached statistical significance, and in other datasets only limited immune subsets were significant. The box-and-whisker plots demonstrate variability, but do not fully support the claim of statistically significant heterogeneity across datasets. The authors should:

• Clarify whether “heterogeneity” refers to statistical significance or visual distribution variability.

• Rephrase the statement to reflect observed trends rather than broadly claiming significant heterogeneity.

• Possibly include variance metrics or formal heterogeneity testing.

The initially submitted manuscript contained an inadequate translation of the phrase “Significant inter- and intratumoral heterogeneity was observed in the CMC immune infiltrate.” Following the recommendations, the interpretation of the results was clarified in lines 447-450 as follows: “The observed variable behavior and trends for the 22 infiltrating immune cells inferred using CIBERSORTx between CMCs and matched adjacent healthy mammary tissues for each gene expression dataset, suggest a notable inter- and intratumoral heterogeneity in the CMC immune infiltrate.”

b) Survival Analysis Clarification

The Discussion states: ‘Overexpression of 24 DEGs and underexpression of 30 DEGs were significantly linked to OS.’ However, Figure 6 only displays three upregulated and three downregulated genes. To fully support the claim:

• The full list of 54 significant DEGs should be provided in a supplementary table with hazard ratios and confidence intervals.

• Figure 6 panel labels (A and B) should be clearly displayed.

• Clarify whether multiple testing correction was applied in survival analysis.

Initially for the survival analysis, the mean expression of each DEG was calculated and Kaplan-Meier and LogRank test methods were applied to compare the low and high expression survival curves. This resulted in 54 DEGs with a LogRank p-value < 0.05, suggesting that these 54 DEGs were significantly associated with OS. However, multiple testing correction was not applied.

Following the recommendation, the survival analysis was expanded to include a univariate Cox proportional hazards regression model and a multiple testing correction. In Materials and methods section, the following modifications were made:

• In lines 270-271, the phrase “Overall survival (OS) was defined as the time in days from diagnosis to death or last follow-up” was included.

• In lines 273-278, the paragraph “Kaplan-Meier survival plots were generated, and a LogRank p < 0.05 was considered for identifying DEGs with nominal associations with OS. In addition, a univariate Cox proportional hazards regression model was fitted for each gene to estimate the hazard ratio (HR) and its 95% confidence interval (CI). p-values derived from the Cox models were adjusted for multiple testing using the Benjamini–Hochberg method. Genes with significant FDR-adjusted p-values were considered associated with OS” and were included.

According to the proposed modification in the methodology, the results were modified as follows:

• Lines 463-477: “The Cox proportional hazards regression model and Log-rank test shown 25 overexpressed DEGs with nominal associations with survival (LogRank p < 0.05) (STOML1, GADD45A, FAM20A, MIR29B-2, TM4SF5, ITGB2, DHDDS, ZCCHC17, RANBP3, TM4SF4, PSMD4, MFHAS1, S100A14, PIM1, PPBP, SFN, NELFE, KLHL38, SIDT1, VTI1B, SCPEP1, PPM1N, KRT73, SCPEP1, FYN and WASF3) whose higher expression levels were associated with an increased risk of mortality (HR > 1). Conversely, 29 underexpressed DEGs with nominal associations with survival (LogRank p < 0.05) (TRPC1, EMX2, NTSR1, CPXM2, HDGFL2, DDAH2, PRKAG2, TRIOBP, DSG1, ANKRD13A, IGFBP2, FOXP4, DOK1, QKI, MFGE8, CYP26B1, AVEN, NINJ2, ASCL2, SYN2, TMEM252, RRAGB, MPRIP, SELENOP, G6PC1, TSR3, FBLN1, CCDC152, FAM187A) were associated with a reduced risk of mortality (HR < 1), suggesting a potential protective effect. However, the 95% IC and multiple testing correction using the Benjamini–Hochberg method, shown none of the genes remained statistically significant (FDR > 0.05) (S11 Table), indicating that these findings should be interpreted as exploratory and require validation in larger cohorts. Kaplan-Meier curves of the top 3 upregulated and downregulated DEGs related to OS are shown in Fig 6.”

• The full list of 54 DEGs with HR, IC, p-value and FDR was provided in S11 table.

• Figure 6 panel labels (A and B) was included.

In Discussion section, the following modification was made:

• Lines 594-598: “Survival analysis of the GSE119810 dataset revealed that 25 overexpressed and 29 underexpressed DEGs were nominally linked to OS, indicating that these DEGs might serve as potential prognostic biomarkers for CMC. However, after correction for multiple testing using the Benjamini–Hochberg method, none of the genes remained statistically significant, indicating that these findings should be interpreted as exploratory and require validation in larger cohorts.”

c) Data Availability Statement

While public datasets are clearly referenced, the proprietary CPA-UN dataset does not indicate:

• GEO/SRA accession numbers

• Repository deposition plans

• Raw FASTQ availability

PLOS ONE requires full availability of underlying data. The manuscript must specify where the proprietary dataset will be deposited and provide accession numbers prior to publication.

The raw FASTQ files were submitted to the NCBI Sequence Read Archive (SRA) with the submission code SUB16061762. The BioProject was created with the code PRJNA1440953. Once the article is published, the files will be made publicly accessible.

In lines 144-146, the availability of the dataset was included as follows: “Our dataset was named “CPA-UN” and the corresponding raw RNASeq data are available on the Sequence Read Archive (SRA) section of The National Center for Biotechnology Information (NCBI) platform BioProject PRJNA557680.”

d) Methods Clarification

• Functional enrichment (GO and KEGG) should be clearly described in the Methods section with appropriate citations.

In lines 192-200 the methodology used to perform GO and KEGG functional enrichment was included as follows: “The list of DEGs was used as an input set in both tools and the enrichment of Gene Ontology (GO) terms in the categories Biological Process (BP), Molecular Function (MF) and Cellular Component (CC) was evaluated, as well as the enrichment of metabolic pathways in Kyoto Encyclopedia of Genes and Genomes (KEGG). The analysis was performed using the enrichGO and enrichKEGG functions from the clusterProfiler and Pathview packages. p-values were calculated using hypergeometric tests and adjusted for multiple comparisons using the Benjamini–Hochberg method to control for false discovery rates (FDR). Terms with FDR-adjusted p-values (padj < 0.05) were considered significantly enriched.”

• All bioinformatics tools (R version, packages, command-line tools) require formal citations.

The bioinformatics tools and their version used were properly referenced (references 13-25).

• Clarify normalization approach used before merging datasets for combined GSEA and co-expression network construction.

In lines 205-208 the methodology used to perform normalization in GSEA for CPA-UN dataset was included as follows: “Additionally, a Gene Set Enrichment Analysis (GSEA) was conducted using the raw count matrix from the CPA-UN gene expression dataset, which was normalized with DESeq2 package (version 2.11.40.8) in R (Version 4.2.3) using the median of ratios method to normalize count data and produce a ranked list of DEGs generated by DESeq2 analysis as input for GSEA (Version 4.4.0).”

In lines 214-227 the methodology used to perform the normalization of a single matrix constructed by merging the raw count tables of the 4 expression sets used in the GSEA was included as follows: “A GSEA was also performed, combining the four gene expression data sets (CPA-UN, GSE119810, GSE136197, and GSE135183) into a single matrix. Only genes present across all datasets were retained to ensure comparability. The resulting matrix of raw counts was used as input for downstream normalization and analysis. Normalization was performed using the regularized logarithmic transformation (rlog) method and variance stabilization was performed with DESeq2 package (version 2.11.40.8) in R (Version 4.2.3). Size factors were estimated to account for differences in sequencing depth and library composition among samples, enabling accurate comparison of gene expression levels across the integrated datasets. The DESeq2 normalization framework models count data using a negative binomial distribution and applies internal scaling factors to correct for technical variability. Normalized expression values and differential expression statistics obtained from DESeq2 were subsequently used to construct a ranked list of DEGs as input for GSEA (Version 4.4.0). The molecular signatures affected by the DEGs were identified following the same methodological approach described for the GSEA of the CPA-UN gene expression data set.”

In lines 242-248 the methodology used to perform the normalization of a single matrix constructed by merging the raw count tables of the 4 expression sets and the subsequent co-expression network analysis was included as follows: “Additionally, a co-expression network was created by merging the four gene expression datasets (CPA-UN, GSE119810, GSE136197, and GSE135183) into a single matrix. The merged matrix of raw counts, normalization (rlog method) and variance stabilization were performed using the same methodological approach followed for the GSEA with DESeq2 package (version 2.11.40.8) in R (Version 4.2.3). From the merged matrix of normalized counts, construction and analysis of the co-expression network were performed following the same methodological approach as for the CPA-UN dataset.”

e) Minor Editorial Revisions

• Line 47: Add citation regarding increasing trend of HBC incidence.

In line 67, the citation corresponding to an updated article mentioning the increasing incidence of HBC (reference 4) was included.

• Line 48: Define TNM before first use.

In lines 68-69, TNM was defined in its first use.

• Line 83: change 'y' to 'and'.

In line 85, “y” was changed to “and”,

• Line 102: Table 1, replace 'Si' with 'Yes' for lymph node metastasis.

In line 123, “Si” was changed to “Yes” in Table 1.

• Clarify the term 'small world' in the co-expression network description for non-specialist readers.

In lines 383-384 term “small world” in the co-expression network was described as follows: “possibly a “small-world network”, defined as a network with a high clustering coefficient and low distances between nodes.”

• Screen entire manuscript for minor typographical inconsistencies.

We look forward to hearing from you soon.

Yours sincerely,

Carlos Alberto Parra López, MD. PhD

Attachment Submitted filename: Response to Reviewers.pdf

# Peer review: Decision Letter 1

**DOI:** 10.1371/journal.pone.0354033.r003

**Authors:** Alexis G. Murillo Carrasco

4 May 2026

<div>PONE-D-25-61057R1

Differentially expressed genes with potential diagnostic and prognostic value in canine mammary carcinoma and their relevance for human breast cancer: an integrated bioinformatics analysis

PLOS One

Dear Dr. Parra-López,

Thank you for submitting your manuscript to PLOS ONE. After careful consideration, we feel that it has merit but does not fully meet PLOS ONE’s publication criteria as it currently stands. Therefore, we invite you to submit a revised version of the manuscript that addresses the points raised during the review process.<section data-scroll-anchor="false" data-testid="conversation-turn-4" data-turn="assistant" data-turn-id="request-WEB:ff59f24d-394d-41da-98f7-ef55db59d8eb-1" dir="auto">

==============================

Please address the reviewers’ comments thoroughly and incorporate their suggested revisions into the manuscript where appropriate. In particular, avoid overstating the results and ensure that your interpretations remain aligned with the actual magnitude of the observed effects. I also suggest avoiding overemphasis on the translational relevance to human disease, as this aspect may be more speculative, especially given that the condition has been more comprehensively characterized in humans, including the definition of molecular subtypes, which are not fully represented in the canine model described here.

==============================

Please submit your revised manuscript by Jun 18 2026 11:59PM. If you will need more time than this to complete your revisions, please reply to this message or contact the journal office at plosone@plos.org. When you're ready to submit your revision, log on to https://www.editorialmanager.com/pone/ and select the 'Submissions Needing Revision' folder to locate your manuscript file.

</section>

Please include the following items when submitting your revised manuscript:

- A letter that responds to each point raised by the academic editor and reviewer(s). You should upload this letter as a separate file labeled 'Response to Reviewers'.
- A marked-up copy of your manuscript that highlights changes made to the original version. You should upload this as a separate file labeled 'Revised Manuscript with Track Changes'.
- An unmarked version of your revised paper without tracked changes. You should upload this as a separate file labeled 'Manuscript'.

If you would like to make changes to your financial disclosure, please include your updated statement in your cover letter. Guidelines for resubmitting your figure files are available below the reviewer comments at the end of this letter.

If applicable, we recommend that you deposit your laboratory protocols in protocols.io to enhance the reproducibility of your results. Protocols.io assigns your protocol its own identifier (DOI) so that it can be cited independently in the future. For instructions see: https://journals.plos.org/plosone/s/submission-guidelines#loc-laboratory-protocols. Additionally, PLOS ONE offers an option for publishing peer-reviewed Lab Protocol articles, which describe protocols hosted on protocols.io. Read more information on sharing protocols at https://plos.org/protocols?utm_medium=editorial-email&utm_source=authorletters&utm_campaign=protocols.

As the corresponding author, your ORCID iD is verified in the submission system and will appear in the published article. PLOS supports the use of ORCID, and we encourage all coauthors to register for an ORCID iD and use it as well. Please encourage your coauthors to verify their ORCID iD within the submission system before final acceptance, as unverified ORCID iDs will not appear in the published article. *Only*  the individual author can complete the verification step; PLOS staff *cannot*  verify ORCID iDs on behalf of authors.

We look forward to receiving your revised manuscript.

Kind regards,

Alexis G. Murillo Carrasco

Academic Editor

PLOS One

**Journal Requirements:**

If the reviewer comments include a recommendation to cite specific previously published works, please review and evaluate these publications to determine whether they are relevant and should be cited. There is no requirement to cite these works unless the editor has indicated otherwise.

[Note: HTML markup is below. Please do not edit.]

Reviewers' comments:

Reviewer's Responses to Questions

**Comments to the Author**

1. If the authors have adequately addressed your comments raised in a previous round of review and you feel that this manuscript is now acceptable for publication, you may indicate that here to bypass the “Comments to the Author” section, enter your conflict of interest statement in the “Confidential to Editor” section, and submit your "Accept" recommendation.

Reviewer #1: All comments have been addressed

Reviewer #2: All comments have been addressed

Reviewer #3: (No Response)

Reviewer #4: (No Response)

**********

2. Is the manuscript technically sound, and do the data support the conclusions?

The manuscript must describe a technically sound piece of scientific research with data that supports the conclusions. Experiments must have been conducted rigorously, with appropriate controls, replication, and sample sizes. The conclusions must be drawn appropriately based on the data presented.

Reviewer #1: Partly

Reviewer #2: Partly

Reviewer #3: Partly

Reviewer #4: No

**********

3. Has the statistical analysis been performed appropriately and rigorously?

Reviewer #1: Yes

Reviewer #2: Yes

Reviewer #3: No

Reviewer #4: No

**********

4. Have the authors made all data underlying the findings in their manuscript fully available?

The PLOS Data policy requires authors to make all data underlying the findings described in their manuscript fully available without restriction, with rare exception (please refer to the Data Availability Statement in the manuscript PDF file). The data should be provided as part of the manuscript or its supporting information, or deposited to a public repository. For example, in addition to summary statistics, the data points behind means, medians and variance measures should be available. If there are restrictions on publicly sharing data—e.g. participant privacy or use of data from a third party—those must be specified.

Reviewer #1: Yes

Reviewer #2: Yes

Reviewer #3: Yes

Reviewer #4: Yes

**********

5. Is the manuscript presented in an intelligible fashion and written in standard English?

PLOS ONE does not copyedit accepted manuscripts, so the language in submitted articles must be clear, correct, and unambiguous. Any typographical or grammatical errors should be corrected at revision, so please note any specific errors here.

Reviewer #1: Yes

Reviewer #2: No

Reviewer #3: Yes

Reviewer #4: Yes

**********

6. Review Comments to the Author

Please use the space provided to explain your answers to the questions above. You may also include additional comments for the author, including concerns about dual publication, research ethics, or publication ethics. (Please upload your review as an attachment if it exceeds 20,000 characters)

**Reviewer #1:**  The revised manuscript is clearly improved and much easier to follow compared to the previous version. The authors have made a genuine effort to address key reviewer concerns, particularly by refining the interpretation of immune infiltrate results, expanding the survival analysis, and improving the clarity of the methods section. The addition of Cox proportional hazards modeling and multiple testing correction is an important step forward and strengthens the overall analytical framework.

That said, I have selected “Yes” where required by the review form; however, some aspects are more accurately considered partially addressed, as noted below.

From a technical standpoint in response to Q2, while the study is generally well designed and uses appropriate and widely accepted bioinformatics tools, there are still limitations that affect how strongly the conclusions can be supported. In particular, the relatively small size of the proprietary dataset (n=10) and the reliance on integrated public datasets introduce variability and limit robustness. In addition, some of the biological conclusions especially around prognostic biomarkers are still somewhat stronger than what the data can fully support at this stage.

Regarding statistical analysis in response to Q3., the revisions are appreciated and clearly move in the right direction. However, after applying multiple testing correction, none of the identified genes remain statistically significant. This is an important point and should be consistently emphasized throughout the manuscript. While it is acceptable to present these findings as exploratory, the language should remain cautious and avoid implying validated prognostic significance. For this reason, I consider the statistical component partly accomplished acceptable if the interpretation is clearly aligned with these limitations.

For data availability in response to Q4, the authors have taken steps to deposit the dataset, which is commendable. However, there appears to be some inconsistency in the reported BioProject accession numbers, and it is not entirely clear whether the data will be fully accessible without restriction at the time of publication. This should be clarified to ensure full compliance with PLOS data sharing requirements.

On a positive note, the manuscript is now well written and generally clear, with improved organization and fewer ambiguities in terminology and interpretation.

In summary, the manuscript is technically solid and suitable for publication in principle, but with the expectation that the authors make final adjustments to:

(1) further temper conclusions especially regarding prognostic biomarkers,

(2) clearly and consistently frame survival results as exploratory, and

(3) finalize and clarify data availability details.

With these points addressed, the manuscript should be acceptable.

**Reviewer #2:** The revision appears thorough and responsive. However, the title, abstract and some discussion seem to overstate their findings.

1. The title may be changed/shortened to "Diagnostic and prognostic values of differentially expressed genes in canine mammary carcinoma: an integrated bioinformatics analysis"

2. The abstract and discussion should restrain from stating too much human relevance due to the lack of substantial data.

3. The abstract's introduction should be shortened and clearly state its aims, while the results section should be expanded.

**Reviewer #3:**  Dear authors,

This study presents an integrated bioinformatics analysis of RNA-seq datasets to identify differentially expressed genes (DEGs) with potential diagnostic and prognostic relevance in canine mammary carcinoma (CMC). By combining one proprietary dataset with three publicly available datasets, the aim is to identify shared transcriptional signatures between tumor and adjacent normal tissues, explore functional enrichment, infer immune infiltration, construct co-expression networks and assess associations with overall survival.

The study addresses a relevant topic within comparative oncology and leverages multiple publicly available datasets, which is a strength. The multi-layered analytical approach, including differential expression, enrichment analysis, network construction, and survival analysis, is also optimal. However, several methodological limitations and inconsistencies reduce the robustness of the conclusions. In particular, the lack of batch effect correction during dataset integration, potential biases in immune deconvolution, and the overinterpretation of exploratory findings impact the reliability of the results. Additionally, some claims, especially regarding translational relevance to human breast cancer and the identification of biomarkers, appear overstated relative to the data presented.

Major comments:

1.Overstatement of translational relevance (Title and throughout the manuscript):

The manuscript suggests direct relevance to human breast cancer; however, no cross-species validation or integrative analysis with human datasets is performed. The connections to human breast cancer rely primarily on previously published literature. This overstates the translational contribution and should be moderated.

2.Lack of batch effect correction in multi-dataset integration:

A central limitation of the study is the integration of multiple RNA-seq datasets without explicit batch effect correction. This issue affects several downstream analyses, including DEG identification, GSEA, co-expression network construction, and immune deconvolution. Given the heterogeneity across datasets, uncorrected technical variability may significantly bias the results and lead to misleading biological interpretations.

3.DEG filtering strategy and exclusion of non-coding genes:

The exclusion of genes with very high counts is not standard practice and lacks justification, potentially removing biologically relevant signals. Additionally, the removal of non-coding genes limits the scope of the analysis by excluding important regulatory elements such as polyadenylated lncRNAs.

4.Immune infiltrate deconvolution limitations:

The use of the LM22 signature matrix, derived from human data, may introduce bias when applied to canine samples. Furthermore, multiple statistical comparisons were performed without clear correction for multiple testing, increasing the risk of false-positive findings. Many reported observations are based on non-significant trends, which should be interpreted cautiously.

5.Co-expression network analysis concerns:

The construction of co-expression networks without correcting for batch effects is particularly problematic, as correlation-based methods are highly sensitive to technical variation. Restricting the network to DEGs may also bias hub gene identification. Additionally, the classification of networks as “small-world” is not sufficiently supported without comparison to appropriate null models. Inconsistencies in reporting (e.g., “top 10” hub genes including more than 10 genes) further reduce clarity.

6.GSEA and functional enrichment interpretation:

While enrichment analyses identify relevant biological processes, the integration of multiple datasets without correction may bias GSEA results. The interpretation would benefit from a more integrated and critical discussion rather than descriptive reporting.

7.Survival analysis limitations:

The survival analysis is limited by a relatively small sample size (n = 47), which likely reduces statistical power. Although several genes show nominal significance, none remain significant after multiple testing correction, suggesting a high likelihood of false-positive findings. These results should be clearly framed as exploratory, and the potential for overfitting should be addressed.

8.Overinterpretation of candidate biomarkers:

The identification of seven shared DEGs is interesting; however, given the limited overlap across heterogeneous datasets and absence of experimental validation, their roles as “diagnostic” or “crucial” biomarkers are overstated. These should be presented as candidate genes requiring further validation.

9.Integration of results in the discussion:

Although the discussion attempts to integrate multiple analytical layers, it does not sufficiently account for dataset variability and methodological limitations. This leads to an overextended interpretation of findings, particularly regarding immune infiltration patterns and pathway activation.

Minor comments:

- Introduction - clarity of novelty:

The introduction would benefit from a clearer definition of the specific knowledge gap addressed by this study and how it advances beyond existing transcriptomic analyses.

- Methods - sample handling details:

Additional details regarding tissue fragment size and RNAlater volume relative to tissue mass would improve reproducibility.

- RNA-seq pipeline – strandedness:

The absence of explicit strandedness parameters during quantification may affect gene-level accuracy, particularly for overlapping transcripts.

- DEG variability across datasets:

The large variation in the number of DEGs identified across datasets should be discussed more explicitly, as it may reflect technical or biological heterogeneity.

- Functional enrichment presentation:

Results are largely presented as lists of enriched terms; a more synthesized biological interpretation would improve readability and impact.

- Downregulated gene interpretation:

The enrichment of muscle-related processes among downregulated genes is biologically meaningful but insufficiently explored.

- Redundancy in GSEA results:

Some pathways are reported multiple times and should be consolidated for clarity.

- Immune infiltrate interpretation:

Non-significant findings should be clearly distinguished from statistically supported results to avoid overinterpretation.

- Reporting inconsistencies:

There are inconsistencies in reporting hub genes (e.g., “top 10” lists exceeding 10 genes) and minor issues such as duplicated gene names that should be corrected.

- Conclusion – tone adjustment:

The conclusions should be moderated to reflect the exploratory nature of the findings and the lack of validation, particularly regarding biomarker claims and relevance to human breast cancer.

In summary, this study addresses an important topic in comparative oncology and presents a multi-layered bioinformatics analysis that has the potential to contribute to the understanding of canine mammary carcinoma. However, several methodological limitations (particularly related to dataset integration, lack of batch effect correction, and the exploratory nature of some analyses) reduce the robustness of the conclusions. In addition, certain interpretations, especially regarding biomarker identification and translational relevance to human breast cancer, appear to be overstated relative to the supporting evidence. Addressing these points and moderating the claims would substantially strengthen the scientific rigor, clarity, and overall impact of the manuscript.

**Reviewer #4:**  Abstract requires major revision. There is no clear description of RNAseq data CPA-UN, which is confusing and lacks transparency. A vague and brief reference to “a proprietary dataset” is not sufficient.

The terminology over/under expressed is technically inaccurate and should be changed to reflect the comparative outcome that is being measured, i.e. transcript levels were higher or lower in one group compared to another.

I agree with the previous reviewer that using the term heterogeneity is confusing when no distribution analyses between studies were completed or differences between groups have been found. Variation within and between would be expected. Additionally, the variation is not likely all biological. There are likely components due to sample collection, handling, and preservation, etc.

The conclusion statement should be adjusted to reflect these results require further investigation before any definitive interpretations can be made. Furthermore, the limitations of secondary data analysis and likely contributions tissue collection and handling issues should be added.

Introduction

Brief and concise. Some of the ideas/topics would fit better in the discussion section. What is meant by “close relationship” between dogs and humans should be more clearly defined.

Methods

Methods are inclusive of info required for this work to be reproduced. This section could be organized better. Furthermore, additional consideration toward data cleaning may impact the outcome of your work.

Data handling/cleaning is not clear. There was quality control of RNA; however, it was not stated if data were cleaned to remove tissue specific genes that do not belong in mammary glands i.e. muscle tissue, skin tissue, which are easy to contaminate MG samples. This is highly relevant to the outcome of this study, as the results suggest problems with data quality.

More compelling rationale must be provided for including the data set GSE135183 as this RNAseq project used RNA extracted from stroma excised by LCMD from FFPE tissues. All other RNAseq projects included in this study used frozen carcinoma tissues, therefore, the data in GSE135183 are from a tissue subtype, not whole tissue. Furthermore, the tissues were preserved differently. This must be considered and put into context.

More compelling rationale for integrating versus analyzing data separately should be provided either in the methods or results section.

I suggest a separate data handling and statistical analysis section.

Results

Pathway analysis results represented in figure 2 are misreported in the results section. Results in Figure 2a strongly suggest that at least some of the carcinoma tissue samples were contaminated with muscle. Nine out of ten biological processes listed are muscle associated. The text of the results section reports only nine “top 10 enriched and significant biological process” and none of the ten categories listed in figure 2a when referring to “significant biological process” on lines 314-319.

Figure 1, fyi, excluding GSE135183 adds three more increased expression genes to the “in common with all data sets” category. One of these, MIA, is an interesting cancer gene.

Pvalue rankings and color associations are inconsistent across Figure 2 panels.

Figure 2 contains results of downregulated DEGs. In other words, Figures 2 and S1 contain the same results!

Figure S1 panels in the figure and those indicated in the corresponding legend disagree.

Line 358, “E2F targets” is repeated

Line 361, “glycolysis” is not evident in figure 3.

Figures 3, 4, Compelling rationale for analyzing CPA-UN separately and then integrated with the three GSE datasets was not provided.

Figure 5 A is null data and therefore not necessary. In the corresponding text: lines 427-430 it is reported that there were no significant differences. This should be the end of the results story here; therefore, it is inappropriate to report lines 430 – 433 that there was an increase in M1, NKs, and plasma, and a decrease M2, CD4 and CD8 Ts. This must be removed. It would be appropriate to present these trends in the discussion section as a comparison with the other RNAseq data sets used.

Figure 5B, the asterisks indicating significant differences for Tregs was difficult to see and should be moved or made larger. Same for Figure S8.

Line 455, a Student’s t test here is not conservative and does not correct for multiple comparisons. This should be an ANOVA followed by an appropriate post hoc test to compare groups if the F test is significant. Alternatively, it may be more appropriate to use a nonparametric statistical method here. Same for Figure S8.

Compelling rationale for analyzing all datasets separately for deconvolution analysis needs to be provided.

Lines 473 – 477, if none of these gene differences were statistically significant after the appropriate statistical testing procedure, they should not be presented as significantly different in Figure 6.

Lines 476, 480, 481, a more detailed description of how the “top” genes were selected for figure 6 is required.

Discussion section:

The discussion lacks depth and impact.

Much of the first paragraph of the discussion section is introductory material and could be removed or moved to the introduction.

Repetition of the methods and results makes up too much of the discussion section. The genes

Lines 691-692, I suggest changing the wording or presentation here. There is no real clinical value in gene expression signatures as potential diagnostic biomarkers for tumor bearing animals.

More discussion regarding the nonoverlap between RNAseq database used is warranted as this work highlights the need for robust and rigorous studies to gain reliable data and information regarding CMC.

Discussion of the biopsy and sample handling differences in the context of this and future studies is required. The value this study is that it highlights the problems of working with mammary carcinoma tissue, the limitations of small sample sizes, and an alarming level of nonoverlap between studies.

**********

7. PLOS authors have the option to publish the peer review history of their article (what does this mean?). If published, this will include your full peer review and any attached files.

If you choose “no”, your identity will remain anonymous but your review may still be made public.

Do you want your identity to be public for this peer review? For information about this choice, including consent withdrawal, please see our Privacy Policy.

Reviewer #1: **Yes:**  Farruk Kabir

Reviewer #2: No

Reviewer #3: No

Reviewer #4: No

**********

[NOTE: If reviewer comments were submitted as an attachment file, they will be attached to this email and accessible via the submission site. Please log into your account, locate the manuscript record, and check for the action link "View Attachments". If this link does not appear, there are no attachment files.]

To ensure your figures meet our technical requirements, please review our figure guidelines: https://journals.plos.org/plosone/s/figures

You may also use PLOS’s free figure tool, NAAS, to help you prepare publication quality figures: https://journals.plos.org/plosone/s/figures#loc-tools-for-figure-preparation.

NAAS will assess whether your figures meet our technical requirements by comparing each figure against our figure specifications.

# Author comment: Author response to Decision Letter 2

**DOI:** 10.1371/journal.pone.0354033.r004

15 Jun 2026

Bogotá, June 14th, 2026

REVIEWERS

PLOSOne

Dear Reviewers

We are very grateful for the feedback you provided on the submitted manuscript titled " Diagnostic and prognostic values of differentially expressed genes in canine mammary carcinoma: An integrated bioinformatics analysis”.

Below, we provide clarifications, in response to each point you raised.

REVIEWER #1:

In summary, the manuscript is technically solid and suitable for publication in principle, but with the expectation that the authors make final adjustments to:

1. Further temper conclusions especially regarding prognostic biomarkers.

2. Clearly and consistently frame survival results as exploratory.

3. Finalize and clarify data availability details.

With these points addressed, the manuscript should be acceptable.

Dear reviewer, we sincerely thank you for carefully evaluating our manuscript and for recognizing its overall technical quality. We appreciate your constructive recommendations. We have addressed each point and included our responses in the revised manuscript. We also appreciate the reviewer’s thoughtful comments, which we believe have significantly improved the manuscript's clarity, balance, and overall rigor.

1. We further moderated our conclusions throughout the manuscript, particularly regarding prognostic biomarkers. Genes identified in the analyses are now consistently described as “candidate biomarkers” or “potential biomarkers” requiring independent validation, rather than definitive markers (lines 68-73 and 824-857).

2. We agree that the survival analyses should be interpreted cautiously. Accordingly, the Results and Discussion sections were revised to clearly and consistently present the survival findings as exploratory given the relatively small sample size and the lack of statistical significance after multiple-testing correction. We also explicitly acknowledge the potential risk of false-positive findings and overfitting (lines 583-586 and lines 779-805).

3. The Data Availability Statement was revised and clarified. The raw FASTQ files have been submitted to the NCBI Sequence Read Archive (SRA) under BioProject accession PRJNA1440953 (submission code: SUB16061762). We clarified that the data will be publicly available upon publication of the manuscript.

REVIEWER #2:

The revision appears thorough and responsive. However, the title, abstract and some discussion seem to overstate their findings.

1. The title may be changed/shortened to "Diagnostic and prognostic values of differentially expressed genes in canine mammary carcinoma: an integrated bioinformatics analysis".

2. The abstract and discussion should restrain from stating too much human relevance due to the lack of substantial data.

3. The abstract's introduction should be shortened and clearly state its aims, while the results section should be expanded.

We sincerely thank these constructive comments and suggestions. We have addressed all points in the revised version.

1. We appreciate the proposed title change. The title was revised to: “Diagnostic and prognostic values of differentially expressed genes in canine mammary carcinoma: An Integrated bioinformatics analysis” (lines 1-3).

2. We agree that the original manuscript overemphasized its relevance to human breast cancer. According, the abstract and discussion were revised to temper statements about translational applicability and relevance to human disease. References to human breast cancer are now presented primarily in the context of comparative oncology and previously published literature rather than as direct conclusions drawn from this study (lines 28-73 and 600-821).

3. Following your recommendation, the Abstract was reorganized. The introductory section was shortened and now more clearly states the study aim. In addition, the Results section was expanded to provide a clearer summary of the principal findings, including differential expression analysis, pathway enrichment, immune infiltrate deconvolution, co-expression network, and potential candidate biomarkers (lines 28-73).

REVIEWER #3:

We sincerely thank the careful and thorough review of our manuscript and appreciate each of your constructive comments and suggestions. We have addressed all your points in the revised version.

Major comments:

1. Overstatement of translational relevance (Title and throughout the manuscript):

The manuscript suggests direct relevance to human breast cancer; however, no cross-species validation or integrative analysis with human datasets is performed. The connections to human breast cancer rely primarily on previously published literature. This overstates the translational contribution and should be moderated.

• Our intention was to highlight the potential comparative oncology value of canine mammary carcinoma rather than claim direct equivalence to human disease. In response, we revised the Title (lines 1-3), Abstract (lines 28-73), Introduction (lines 97-101), Discussion (lines 600-821), and Conclusions (lines 824-857) to moderate these statements.

2. Lack of batch effect correction in multi-dataset integration:

A central limitation of the study is the integration of multiple RNA-seq datasets without explicit batch effect correction. This issue affects several downstream analyses, including DEG identification, GSEA, and co-expression network construction. Given the heterogeneity across datasets, uncorrected technical variability may significantly bias the results and lead to misleading biological interpretations.

• Batch correction was performed using ComBat-seq (SVA package), with dataset origin as the batch variable, although this was not explicitly stated in the original manuscript. This information has been added to the Materials and Methods section, and the Discussion was expanded to acknowledge the possibility of residual technical variability despite batch correction (lines 257-260 and 620-623).

3. DEG filtering strategy and exclusion of non-coding genes:

The exclusion of genes with very high counts is not standard practice and lacks justification, potentially removing biologically relevant signals. Additionally, the removal of non-coding genes limits the scope of the analysis by excluding important regulatory elements such as polyadenylated lncRNAs.

• The filtering strategy was revised and clarified in the Materials and methods section. This filtering strategy increased the number of DEGs across all datasets. Nevertheless, the functional enrichment analysis yielded results consistent with those reported using the previous filtering approach. In contrast, the inclusion of LOC genes modified the Results and Discussion of both the co-expression network and survival analyses. S4 Table has also been added, listing the predicted/uncharacterized genes annotated with LOC identifiers and their corresponding gene biotypes, as available in the GTF file (lines 211–213 and 352-356).

4. Immune infiltrate deconvolution limitations:

The use of the LM22 signature matrix, derived from human data, may introduce bias when applied to canine samples. Furthermore, multiple statistical comparisons were performed without clear correction for multiple testing, increasing the risk of false-positive findings. Many reported observations are based on non-significant trends, which should be interpreted cautiously.

• We acknowledge that the LM22 signature matrix was derived from human leukocyte expression profiles and may not fully represent the immune landscape of canine carcinomas. However, due to the limited availability of validated canine-specific reference matrices, LM22 has previously been used as an exploratory approach in canine cancers ((doi: 10.1038/s42003-023-05208-z, doi: 10.3389/fvets.2023.1192525, doi: 10.1016/j.ccell.2020.01.004). Therefore, we applied this method as an exploratory analysis and explicitly instated in that the CIBERSORTx results should be interpreted cautiously as estimates rather than absolute quantifications. This limitation was also addressed in the Discussion section (lines 302-308, 313-318, and 770–777).

• The immune deconvolution analysis in the Methods section was revised to improve statistical rigor. The paired Student’s t test was replaced with the Wilcoxon rank-sum test, and p-values were adjusted using the Benjamini–Hochberg correction. The Results and Discussion sections were revised to distinguish statistically significant findings from non-significant trends, which are now described more cautiously as exploratory observations ((lines 313–321, 530-557, and 752-762).

5. Co-expression network analysis concerns:

The construction of co-expression networks without correcting for batch effects is particularly problematic, as correlation-based methods are highly sensitive to technical variation. Restricting the network to DEGs may also bias hub gene identification. Additionally, the classification of networks as “small-world” is not sufficiently supported without comparison to appropriate null models. Inconsistencies in reporting (e.g., “top 10” hub genes including more than 10 genes) further reduce clarity.

• We appreciate these important methodological observations. As noted above, batch correction was performed using ComBat-seq. Initially, the network was intentionally constructed using DEGs to focus on transcriptional alterations associated with CMC, while recognizing that this approach may influence hub-gene identification and does not capture the full transcriptomic network. We have now adopted an unsupervised, variance-based approach, and the number of genes included in the network was determined using the elbow method applied to the variance distribution. Differential expression results were used only for node annotation and were not involved in network construction or hub gene identification. The term “small-world” was removed from the Results section. The co-expression network analysis was revised to report the network construction strategy, key topological metrics, module-specific hub genes, and co-expression modules. The manuscript was revised to correct reporting inconsistencies, including the inaccurate reference to “top 10” hub genes (lines 275-298 and 465-524).

6. GSEA and functional enrichment interpretation:

While enrichment analyses identify relevant biological processes, the integration of multiple datasets without correction may bias GSEA results. The interpretation would benefit from a more integrated and critical discussion rather than descriptive reporting.

• We thank you for this constructive suggestion. As noted earlier, batch correction was performed before downstream analyses, including GSEA. The discussion was revised to offer a more integrative and critical interpretation of the GSEA results, emphasizing recurrent biological themes across datasets rather than individual enriched pathways. Interpretations were also moderated, and pathway enrichment is now described as transcriptomic patterns rather than direct evidence of functional activation (lines 704-723).

7. Survival analysis limitations:

The survival analysis is limited by a relatively small sample size (n = 47), which likely reduces statistical power. Although several genes show nominal significance, none remain significant after multiple testing correction, suggesting a high likelihood of false-positive findings. These results should be clearly framed as exploratory, and the potential for overfitting should be addressed.

• In the revised manuscript, the survival analysis is now explicitly described as exploratory. The revised gene-filtering strategy altered the number of DEGs included in the analyses, necessitating in corresponding updates to the Results, which have been revised throughout the manuscript. We clarify that although several genes showed nominal associations with survival outcomes, none remained statistically significant after multiple-testing correction. Accordingly, in the Discussion, these genes are now presented as preliminary prognostic candidates requiring validation in larger independent cohorts ((lines 332-339, 565-582, and 779-805).

8. Overinterpretation of candidate biomarkers:

The identification of seven shared DEGs is interesting; however, given the limited overlap across heterogeneous datasets and absence of experimental validation, their roles as “diagnostic” or “crucial” biomarkers are overstated. These should be presented as candidate genes requiring further validation.

• The manuscript has been revised to refer to these genes as “candidate genes” or “candidate biomarkers” rather than validated diagnostic markers. We also emphasize the limited overlap among datasets and the lack of experimental validation, and we have moderated the conclusions accordingly (lines 600-857).

9. Integration of results in the discussion:

Although the discussion attempts to integrate multiple analytical layers, it does not sufficiently account for dataset variability and methodological limitations. This leads to an overextended interpretation of findings, particularly regarding immune infiltration patterns and pathway activation.

• The Discussion was revised to more explicitly acknowledge dataset variability and methodological limitations throughout the interpretation of the results. We moderated the discussion of immune infiltration patterns, pathway enrichment, and candidate genes, emphasizing their exploratory nature and the need for further validation (lines 600-821).

Minor comments:

- Introduction - clarity of novelty:

The introduction would benefit from a clearer definition of the specific knowledge gap addressed by this study and how it advances beyond existing transcriptomic analyses.

• We thank you for this insightful comment. The Introduction was revised to more clearly define the specific knowledge gap this study addresses and to better highlight its novelty. A paragraph addressing this suggestion was added (lines 103-108). Additionally, the introduction was enhanced with further suggestions from reviewer #4.

- Methods - sample handling details:

Additional details regarding tissue fragment size and RNAlater volume relative to tissue mass would improve reproducibility.

• To improve methodological clarity and reproducibility, additional details on tissue handling and preservation were added to into the Materials and methods section (lines 135-142).

- RNA-seq pipeline – strandedness:

The absence of explicit strandedness parameters during quantification may affect gene-level accuracy, particularly for overlapping transcripts.

• Library preparation was performed using the TruSeq Stranded Total RNA Sample Preparation Kit, which generates strand-specific libraries. Accordingly, the appropriate strandedness parameters used during quantification have now been explicitly include in the Methods section to improve transparency and reproducibility (lines 200-202).

- DEG variability across datasets:

The large variation in the number of DEGs identified across datasets should be discussed more explicitly, as it may reflect technical or biological heterogeneity.

We appreciate this important observation. The revised Discussion now acknowledges that the variability in the number of DEGs across datasets likely reflects both biological variability and technical differences among studies, including sample collection, tissue handling, RNA quality, sequencing protocols, and bioinformatic workflows (lines 600-821).

- Functional enrichment presentation:

Results are largely presented as lists of enriched terms; a more synthesized biological interpretation would improve readability and impact.

• In the Discussion section, a paragraph was added to revise the functional enrichment results providing a more integrated biological interpretation rather than primarily listing significantly enriched terms and pathways (lines 674-702).

- Downregulated gene interpretation:

The enrichment of muscle-related processes among downregulated genes is biologically meaningful but insufficiently explored.

• In the Discussion section, a paragraph was added to expand in which the interpretation of the downregulated gene enrichment results (lines 687-698).

- Redundancy in GSEA results:

Some pathways are reported multipl

Attachment Submitted filename: Response_to_Reviewers_auresp_2.pdf

# Peer review: Decision Letter 2

**DOI:** 10.1371/journal.pone.0354033.r005

**Authors:** Alexis G. Murillo Carrasco

3 Jul 2026

Diagnostic and prognostic values of differentially expressed genes in canine mammary carcinoma: An integrated bioinformatics analysis.

PONE-D-25-61057R2

Dear Dr. Parra-López,

We’re pleased to inform you that your manuscript has been judged scientifically suitable for publication and will be formally accepted for publication once it meets all outstanding technical requirements.

Within one week, you’ll receive an e-mail detailing the required amendments. When these have been addressed, you’ll receive a formal acceptance letter and your manuscript will be scheduled for publication.

An invoice will be generated when your article is formally accepted. Please note, if your institution has a publishing partnership with PLOS and your article meets the relevant criteria, all or part of your publication costs will be covered. Please make sure your user information is up-to-date by logging into Editorial Manager at Editorial Manager® and clicking the ‘Update My Information' link at the top of the page. For questions related to billing, please contact billing support.

If your institution or institutions have a press office, please notify them about your upcoming paper to help maximize its impact. If they’ll be preparing press materials, please inform our press team as soon as possible -- no later than 48 hours after receiving the formal acceptance. Your manuscript will remain under strict press embargo until 2 pm Eastern Time on the date of publication. For more information, please contact onepress@plos.org.

Kind regards,

Alexis G. Murillo Carrasco

Academic Editor

PLOS One

Additional Editor Comments (optional):

Reviewers' comments:

Reviewer's Responses to Questions

**Comments to the Author**

1. If the authors have adequately addressed your comments raised in a previous round of review and you feel that this manuscript is now acceptable for publication, you may indicate that here to bypass the “Comments to the Author” section, enter your conflict of interest statement in the “Confidential to Editor” section, and submit your "Accept" recommendation.

Reviewer #2: All comments have been addressed

Reviewer #3: All comments have been addressed

Reviewer #4: All comments have been addressed

**********

2. Is the manuscript technically sound, and do the data support the conclusions?

The manuscript must describe a technically sound piece of scientific research with data that supports the conclusions. Experiments must have been conducted rigorously, with appropriate controls, replication, and sample sizes. The conclusions must be drawn appropriately based on the data presented.

Reviewer #2: Yes

Reviewer #3: Yes

Reviewer #4: Yes

**********

3. Has the statistical analysis been performed appropriately and rigorously?

Reviewer #2: Yes

Reviewer #3: Yes

Reviewer #4: Yes

**********

4. Have the authors made all data underlying the findings in their manuscript fully available?

The PLOS Data policy requires authors to make all data underlying the findings described in their manuscript fully available without restriction, with rare exception (please refer to the Data Availability Statement in the manuscript PDF file). The data should be provided as part of the manuscript or its supporting information, or deposited to a public repository. For example, in addition to summary statistics, the data points behind means, medians and variance measures should be available. If there are restrictions on publicly sharing data—e.g. participant privacy or use of data from a third party—those must be specified.

Reviewer #2: Yes

Reviewer #3: Yes

Reviewer #4: Yes

**********

5. Is the manuscript presented in an intelligible fashion and written in standard English?

PLOS ONE does not copyedit accepted manuscripts, so the language in submitted articles must be clear, correct, and unambiguous. Any typographical or grammatical errors should be corrected at revision, so please note any specific errors here.

Reviewer #2: Yes

Reviewer #3: Yes

Reviewer #4: Yes

**********

6. Review Comments to the Author

Please use the space provided to explain your answers to the questions above. You may also include additional comments for the author, including concerns about dual publication, research ethics, or publication ethics. (Please upload your review as an attachment if it exceeds 20,000 characters)

Reviewer #2: All my concerns have been satisfactorily addressed. Therefore, I recommend accepting it as the current form.

Reviewer #3: I would like to thank the authors for their thorough and rigorous revision of the manuscript. They have carefully addressed all the concerns raised in my previous review.

Specifically, the inclusion of the batch effect correction details using ComBat-seq, the implementation of more robust non-parametric statistical methods (Wilcoxon rank-sum test with Benjamini-Hochberg FDR correction) for the immune deconvolution, and the complete overhaul of the co-expression network analysis have significantly enhanced the methodological soundness of the study. Furthermore, the tone regarding the translational relevance to human disease and the prognostic potential of the candidate biomarkers has been appropriately moderated throughout the text.

The reporting inconsistencies and figure duplicates have also been successfully resolved. In my view, the authors have fully answered all questions, and the manuscript is now sound and suitable for publication.

Reviewer #4: all concerns and suggestions addressed; thank you for completing and sharing your work with the scientific community

Continued use of LCMD mammary carcinoma stroma data GSE135183 in individual and the rational for combining these data with the other datasets is not compelling; however, a more transparent description of the process/methods is included in R2. An improvement in analyzing datasets independently has been incorporated. For the integrated analysis, in my opinion combining the 3 datasets from mammary carcinoma tissue and comparing to the LCMD stromal tissue would have been more biologically relevant.

More DEGs were listed in R2 (N=241) compared to R1 (N=181) in the CPA=UN data

1759 vs 1009 in GSE119810

748 vs 620 in GSE136197

1077 vs 972 in GSE135183

Interestingly, IGFBP5 is an additional gene added to the increased DEGs

using upregulated and downregulated genes terminology is better than over/under expressed

discussion section is still long, however, it is much improved...

**********

7. PLOS authors have the option to publish the peer review history of their article (what does this mean?). If published, this will include your full peer review and any attached files.

If you choose “no”, your identity will remain anonymous but your review may still be made public.

**Do you want your identity to be public for this peer review?**  For information about this choice, including consent withdrawal, please see our Privacy Policy.

Reviewer #2: No

Reviewer #3: No

Reviewer #4: No

**********

# Editor report: Acceptance letter

**DOI:** 10.1371/journal.pone.0354033.r006

**Authors:** Alexis G. Murillo Carrasco

PONE-D-25-61057R2

PLOS One

Dear Dr. Parra-López,

I'm pleased to inform you that your manuscript has been deemed suitable for publication in PLOS One. Congratulations! Your manuscript is now being handed over to our production team.

At this stage, our production department will prepare your paper for publication. This includes ensuring the following:

* All references, tables, and figures are properly cited

* All relevant supporting information is included in the manuscript submission,

* There are no issues that prevent the paper from being properly typeset

You will receive further instructions from the production team, including instructions on how to review your proof when it is ready. Please keep in mind that we are working through a large volume of accepted articles, so please give us a few days to review your paper and let you know the next and final steps.

Lastly, if your institution or institutions have a press office, please let them know about your upcoming paper now to help maximize its impact. If they'll be preparing press materials, please inform our press team within the next 48 hours. Your manuscript will remain under strict press embargo until 2 pm Eastern Time on the date of publication. For more information, please contact onepress@plos.org.

You will receive an invoice from PLOS for your publication fee after your manuscript has reached the completed accept phase. If you receive an email requesting payment before acceptance or for any other service, this may be a phishing scheme. Learn how to identify phishing emails and protect your accounts at https://explore.plos.org/phishing.

If we can help with anything else, please email us at customercare@plos.org.

Thank you for submitting your work to PLOS ONE and supporting open access.

Kind regards,

PLOS ONE Editorial Office Staff

on behalf of

Dr. Alexis G. Murillo Carrasco

Academic Editor

PLOS One
